# The Transcriptomic and Proteomic Molecular Signatures of Equine Multiple-System Neuropathy (Grass Sickness)

**DOI:** 10.3390/cells15151328

**Published:** 2026-07-24

**Authors:** Kim M. Summers, Anna E. Karagianni, Paula Ledesma Fernandez, Philippa M. Beard, R. Scott Pirie, John A. Keen, Thomas M. Wishart, Bruce C. McGorum

**Affiliations:** 1 Mater Research Institute-University of Queensland, Woolloongabba, QLD 4102, Australia; 2 School of Veterinary Medicine, University of Surrey, Guildford GU2 7AL, UK; a.karagianni@surrey.ac.uk; 3 School of Biodiversity, One Health and Veterinary Medicine, College of Medical, Veterinary and Life Sciences, University of Glasgow, Gilmorehill Campus, Glasgow G12 8QQ, UK; paula.ledesmafernandez@glasgow.ac.uk; 4 The Roslin Institute and Royal (Dick), School of Veterinary Studies, University of Edinburgh, Easter Bush, Midlothian EH25 9RG, UK; p.m.beard@keele.ac.uk (P.M.B.); scott.pirie@ed.ac.uk (R.S.P.); john.keen@ed.ac.uk (J.A.K.); 5 School of Life Sciences, Huxley Building, Keele University, Staffordshire ST5 5BG, UK; 6 School of Science and Technology, Nottingham Trent University, Nottingham NG1 4FQ, UK

**Keywords:** multiple-system neuropathy, grass sickness, superior cervical ganglion, neurotoxicity, neurodegeneration, Equidae, transcriptome, proteome, gene expression

## Abstract

**Highlights:**

**What are the main findings?**

- Neurodegeneration in equine grass sickness is associated with similar transcriptomic and proteomic changes.
- The findings indicate dysregulation of neuronal and mitochondrial function and increased cellular stress and inflammation.

**What are the implications of the main findings?**

- Changes are likely due to the neurotoxicity of pathogen or plant nPLA2 enzymes.
- The study improves understanding of neurotoxic neurodegeneration.

**Abstract:**

Equine grass sickness (EGS or equine dysautonomia) is a predominantly fatal multi-system neuropathy affecting grazing horses, likely caused by a neurotoxic phospholipase A2 (nPLA2) derived from a plant or microorganism. We studied neuronal tissue gene and protein expression patterns in EGS to elucidate the possible mechanisms of neurotoxicity and neurodegeneration. Tissue from the cranial cervical ganglion of eight EGS horses and six controls was examined histologically and used for transcriptomic analysis. These transcriptomic data were compared with previously published EGS-related proteomic datasets from different horses. Results were visualized using the network analysis tool BioLayout and Ingenuity Pathway Analysis. The cranial cervical ganglia from all affected horses showed pathology typical of EGS. They also showed distinct gene and protein expression profiles that were different from the controls. The EGS signature consisted ofreduced expression of genes and proteins involved in neurological function (including ion-channel and synaptic-function genes and genes encoding mitochondrial proteins) and increased expression of genes and proteins indicative of cellular stress, cell death and inflammation. This signature likely reflects more generalized neurodegeneration. This study thus improves our understanding of the molecular changes likely to be associated with a neurotoxic neurodegenerative process.

## 1. Introduction

Equine grass sickness (EGS or equine dysautonomia) is a predominantly fatal multiple-system neuropathy affecting grazing horses. It occurs throughout the UK and Northern Europe with a mortality rate between 50% and 80% [1]. Although there is no equivalent condition in humans, an apparently identical disease occurs in cats, dogs, hares, rabbits, llamas and possibly sheep [2,3,4,5,6,7,8]. The classic pathology in cases of EGS is widespread chromatolysis of enteric, parasympathetic and postganglionic sympathetic neurons, general visceral efferent and general somatic efferent lower motor neurons in the brainstem and general somatic efferent lower motor neurons in the spinal cord [9,10]. The clinical signs of EGS largely reflect dysfunction of enteric and autonomic neurons and include dysphagia, generalized ileus, sweating, salivation, ptosis, tachycardia, and rhinitis sicca, which can range from mild crusting of the mucous membranes of the nasal cavity to severe ulcerative lesions (reviewed in [1]). EGS is subdivided into acute, sub-acute and chronic forms according to the severity and duration of the clinical signs. The acute and sub-acute forms are associated with 100% mortality, whereas the chronic form has a survival rate approximating 50%. While the etiology of EGS remains unconfirmed, recent studies identified ultrastructural abnormalities in the skeletal muscle neuromuscular junctions that are consistent with the actions of a neurotoxic phospholipase A2 (nPLA2), presumably derived from plants or soil or resident intestinal microorganisms [10,11,12].

Ultrastructural findings suggest that the lesion in EGS primarily involves the loss of the Golgi apparatus of specific neuronal populations [13]. We previously reported widespread changes in the proteome of the cranial (superior) cervical ganglion (CCG) of horses with EGS [14], including disruption of pathways involving proteins associated with protein misfolding responses, akin to those identified in human neurodegenerative diseases. Analysis of the proteome shows the functional endpoints of the disease state. Since this can be achieved through transcriptional, translational or post-translational mechanisms, we were interested in determining the gene expression changes associated with terminal neuropathy that would precede these proteomic changes. Hence, the aim of the present study was to evaluate gene expression in the CCG, using a transcriptome-wide approach, to elucidate gene-level responses to the ongoing neurodegenerative condition in EGS. We also updated the previous proteomic data to identify proteins not annotated at the time of the original analysis and compared these results with the transcriptomic analysis. This study offers an opportunity to analyze transcriptomic and proteomic alterations in a naturally occurring neurodegenerative disease.

## 2. Materials and Methods

### 2.1. Samples

Sample collection and analysis was approved by the University of Edinburgh Veterinary Ethical Review Committee. Samples were collected at necropsy from horses that were subjected to euthanasia on humane grounds, by intravenous injection of a combination of quinalbarbitone and cinchocaine (Somulose, Dechra, Shrewsbury, UK), with the horse owners’ informed consent.

For transcriptomic analysis, EGS horses comprised four acute and four sub-acute cases, as categorized previously [15] (*n* = 8; median age 7 years, range 7 months–20 years). A combination of acute and sub-acute cases was selected because there are only minor differences in the clinical features and pathology of these phenotypes; chronic EGS cases were not included because of differences in the pathology and natural history of the condition. EGS horses were euthanized due to disease severity (median time after the first onset of abnormal clinical signs in acute cases was 1.5 days, range 1–2 days; sub-acute cases median 2 days, range 2–5 days; Table 1). EGS was confirmed in all cases by necropsy, including histopathological examination of autonomic ganglia [16]. Controls (*n* = 6; median age 11.5 years, range 6–35 years) were euthanized for reasons other than EGS or any other neurological conditions (Table 1).

CCG samples were collected for analysis because chromatolysis of a high proportion of CCG neurons is a consistent feature of EGS [17]. Peripheral connective and adipose tissue were cleaned from the CCG samples and a 1.5 cm thick cross-sectional slice was taken approximately 1 cm from the end of the ganglion and placed in 10% neutral-buffered formalin for at least 24 h before being processed and embedded in paraffin following routine methods. Five μm thick sections were cut and stained with hematoxylin and eosin. A parallel slice of ganglion was also harvested, the fibrous capsule removed with a scalpel blade, and the ganglion tissue placed into RNAlater RNA stabilization reagent (Qiagen, Manchester, UK) at room temperature prior to freezing at −20 °C (within 21 h). Processing was usually performed within two hours post mortem (maximum 10 h).

### 2.2. RNA Analysis

RNA was extracted by Edinburgh Genomics (now Edinburgh Cliinical Research Facility), University of Edinburgh, UK, from the samples of CCG that had been treated with RNAlater, using a guanidinium thiocyanate phenol chloroform extraction procedure (http://genomics.ed.ac.uk, accessed 31 May 2026). The quality and quantity of RNA was assessed using the TapeStation (Agilent Technologies, Stockport, UK). All samples used in the analysis had an RNA Integrity Number of ≥7. Extracted RNA was applied to the Agilent 4X44K horse array (design ID G2519F-021322) and the intensity of the signal for each probe (reflecting RNA level) measured as per manufacturer’s instructions by Edinburgh Genomics. Quality control and normalization were performed by Edinburgh Genomics using the RMA procedure [18] within the Partek Genomics Suite (v6.6; Partek Incorporated, St Louis, MO, USA), producing relative expression values in arbitrary units (AUs). To assess data quality, evaluate sample relationships, and monitor for potential batch effects or outliers, principal component analysis (PCA) was performed on the normalized data matrix using Partek. The dataset was mean-centered across all samples, and feature scaling was weighted by variance to preserve the biological impact of highly variable transcripts. Dimensionality reduction was achieved via Singular Value Decomposition (SVD), and the first three principal components (PCA #1, PCA #2, and PCA #3) were extracted for 3D visualization.

The probes were annotated initially using the annotation file from Agilent Technologies (dated 18 April 2012). Approximately 30,000 probes were given a non-specific gene name, either an LOC number or the probe ID only. The Ensembl transcript and gene IDs for these probes were then retrieved from the annotation file and run through Ensembl BioMart (http://www.ensembl.org/biomart/martview/, accessed between 17 July 2022 and 21 October 2024) to identify the associated genes. The initial pass looked for Ensembl annotations within the horse genome. These were then added to the annotation file. Any remaining gene descriptions were compared with the human annotations and any gene names retrieved were added to the annotation file. Where a Human Genome Nomenclature Committee accession was available, the record was accessed and any gene name added to the file. In the final dataset 8812 probesets were unannotated and 2023 had only an LOC name, while 32,768 of the 43,603 probesets on the array were allocated a more definitive gene name.

### 2.3. Analysis of Gene Expression Patterns

Initially the expression levels were filtered to remove low-expression probes (where no sample reached an intensity of ≥60 AU). The remaining dataset contained the results for 28,752 probesets. A gene-set enrichment analysis (GSEA; https://www.gsea-msigdb.org/gsea/index.jsp, accessed 1 November 2024) was run on the samples. The merged and filtered intensity file was then prepared for analysis by the network analysis tool BioLayout (http://biolayout.org, v.3.4), which was derived from Biolayout Express3D [19]. A pairwise Pearson correlation matrix was calculated for each pair of probesets on the array as a measure of similarity of expression patterns of the different probesets. Graph layout was performed using a modified Fruchterman–Rheingold algorithm [20] in a 3-dimensional space in which nodes (probesets representing transcripts) were connected by weighted, undirected edges representing correlations above the selected threshold. A correlation cut-off of r = 0.90 was used to construct a probe-to-probe graph containing 25,399 nodes and 2,084,949 edges (correlations ≥ 0.90). The topology of the network contained localized areas of high connectivity and high correlation (groups of genes with similar profiles), determined by clustering using the Markov clustering algorithm (MCL) [21], which has been demonstrated to be one of the most effective graph-based clustering algorithms available [22]. An MCL inflation value of 2.2 was used to determine the granularity of clustering, as it has been shown to be optimal when working with highly structured expression graphs [19]. Clusters were numbered according to their relative size, with the largest cluster being designated cluster001. Genes with transcripts within the increased or decreased clusters were subjected to analysis via DAVID functional annotation (version 6.8; http://david.abcc.ncifcrf.gov, accessed 1 November 2024 [23,24]). The lists of genes in clusters with higher or lower average expression in EGS horses were also submitted to the Ingenuity Pathway Analysis (IPA) software (Ingenuity Systems, Silicon Valley, CA, USA), a program built on a hand-curated data “knowledgebase” (http://www.ingenuity.com/products/ipa, accessed 31 July 2025) [25].

To examine the expression of nuclear and mitochondrial genes encoding mitochondrial proteins, a curated list (MitoCarta3.0) of the mitochondrial proteome containing 1136 proteins [26] was downloaded (https://www.broadinstitute.org/mitocarta/mitocarta30-inventory-mammalian-mitochondrial-proteins-and-pathways, accessed 26 August 2025) and expression levels for all probes corresponding to genes in the revised annotated list were retrieved. A total of 1036 genes encoding proteins of the MitoCarta3.0 list were annotated in the updated list.

### 2.4. Proteomics Analysis

The results from an existing published proteomic dataset of EGS and control CCG samples [14] were used; details of the samples and procedures for protein extraction can be found therein. In summary, CCG samples from six EGS and six control horses were pooled by condition and the protein extracts prepared. Two technical replicates from each pool were subjected to iTRAQ proteomic analysis. For the present study, the raw data from the earlier project were reprocessed using the equine database Uniprot Horse (https://www.uniprot.org/proteomes/UP000002281, accessed 22 February 2022) which is based on the horse genome version EquCab3.0 updated January 2022 (http://www.ensembl.org/Equus_caballus/Info/Annotation, accessed 22 February 2022). This process yielded 1814 proteins in addition to those identified previously [14]. The results were then filtered to include only those proteins with a minimum of two unique peptides and a minimum 20% difference between cases and controls [27,28]. The 4125 unique proteins that passed this filtering were then submitted to IPA for further analysis.

### 2.5. Statistical Analysis

GraphPad Prism (v.9, https://www.graphpad.com/) was used for statistical analysis. Pairwise comparisons were made using Student’s *t*-test with Welch’s correction for unequal standard deviations. * 0.01 < *p* ≤ 0.05; ** 0.001 < *p* ≤ 0.01; *** 0.0001 < *p* ≤ 0.001; **** *p* ≤ 0.0001.

## 3. Results

### 3.1. EGS Horses Demonstrated Histopathology Typical of EGS

**Figure 1:**
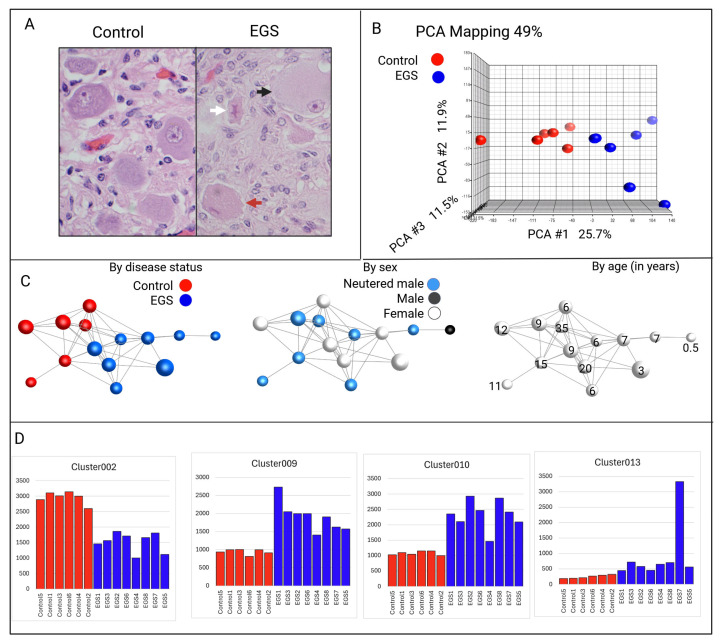
Analysis of CCG samples from control and EGS horses. (**A**) Histology of the cranial (superior) cervical ganglion in EGS. All eight affected horses in the transcriptomic study showed neurodegeneration typical of EGS. The panel on the left is from a control horse and shows normal, healthy neuronal cell bodies. The panel on the right is from a horse with EGS. The cell body of three neurons can be seen in this image. Black arrow—swollen neuronal cell body with loss of the normal speckled basophilic appearance of the cytoplasm (this is known as loss of Nissl substance, or chromatolysis); the nucleus of this neuron is located near the edge of the cell body (peripheralized). White arrow—shrunken neuronal cell body, particularly the nucleus (pyknosis). Red arrow—neuronal cell body with a peripheralized nucleus and clear vacuoles visible in the cytoplasm. (**B**) Principal component analysis of study samples based on gene expression. Analysis including six controls (red) and eight affected horses (blue). Control and EGS horses were clearly separated in component PCA #1. (**C**) Sample-to-sample network for control and EGS horses. Left panel—colored by disease status (control—red; EGS—blue); middle panel—colored by sex (male—black; neutered male—blue; female—white); right panel—age of horses in years is indicated. (**D**) Clusters of co-expressed genes showing different expression patterns. RNA samples from the CCG of 6 control horses and 8 horses with grass sickness were analyzed using the tool BioLayout. A pairwise Pearson correlation coefficient was calculated for every pair of transcripts represented on the array. Using a threshold of r = 0.9, a network graph was constructed, comprising 24,829 nodes (transcripts), connected by 2,096,475 edges. The graph was then clustered using the Markov clustering algorithm with an inflation value (which controls the granularity of clustering) of 2.2. This resulted in 1056 clusters containing ≥ 5 nodes. Shown are the average expressions of transcripts (probes) in the cluster for each sample. The Y-axis shows the normalized intensity; the X-axis shows each horse with the six controls at the left (red) and the eight EGS horses to the right (blue). Only clusters containing more than 100 probes are shown. Cluster002 was the largest downregulated cluster while cluster009 and cluster010 were the largest upregulated clusters. Cluster013 is included because it was upregulated in all EGS horses but particularly in one individual.

Previous studies have shown that at the terminal stage of the disease the majority of CCG neurons are undergoing acute neurodegeneration, although there is a spectrum of stages of neurodegeneration present, ranging from neurons with relatively normal morphology to those in the late stages of acute neurodegeneration [13,14]. Examination of the CCG of all eight EGS horses in the transcriptomic study identified neurodegeneration typical of EGS, characterized by neuronal swelling, cytoplasmic vacuolation, extensive chromatolysis with loss of Nissl substance, and peripheralization and/or pyknosis of the nuclei (Figure 1A). Occasional neuronophagia was also present. In contrast, no significant lesions were present in the CCG of the control horses (Figure 1A). This was consistent with the findings for the horses used in the previous proteomics study [14].

### 3.2. EGS Horses Had Distinct Gene Expression Profiles Compared with Control Horses

There were approximately 44,000 probes on the Agilent Horse Array. Principal component analysis based on the expression data for all probes showed clear segregation of EGS and control horses in PCA #1, explaining 25.7% of the variance (Figure 1B). This indicated a distinctive expression signature associated with EGS, although each group was apparently diverse. Sample-to-sample network analysis using the tool BioLayout with a correlation coefficient threshold of R ≥ 0.93 supported this separation of samples from affected and unaffected animals (Figure 1C) and showed that neither age nor sex was a major component in partitioning the samples.

### 3.3. GSEA Revealed Downregulation of Neurological Function and Upregulation of Stress Responses and Inflammation Markers in EGS Horses

The GSEA showed that the control samples were enriched relative to EGS samples for expression of genes associated with oxidative phosphorylation, fatty-acid metabolism, adipogenesis, myogenesis and angiogenesis. The EGS samples were enriched for expression of genes associated with stress responses, apoptosis and inflammation. The results of the GSEA analysis are available in Table S1.

### 3.4. Network Cluster Analysis Confirmed the Results of the GSEA

To understand in more detail the gene expression patterns associated with terminal EGS, we adopted a network community discovery approach (reviewed in [29]). The network visualization tool BioLayout was used to create a probeset-to-probeset network graph and to group transcripts based on their expression patterns. All probesets representing transcripts with normalized intensity of ≥60 AU in at least one sample were included in the analysis (28,752 probesets). The average expression profiles of the largest up- and downregulated clusters are shown in Figure 1D and a full list of the clusters with average expression profiles is given in Table S2. Strikingly (and consistent with the preliminary PCA results and the sample-to-sample analysis), each horse also showed elevation of a unique subset of transcripts that were at a low (or lower) level in all other horses, independent of EGS status (for example, cluster013 in Figure 1D).

### 3.5. Genes Related to Neurological Function Were Downregulated in EGS

**Figure 2:**
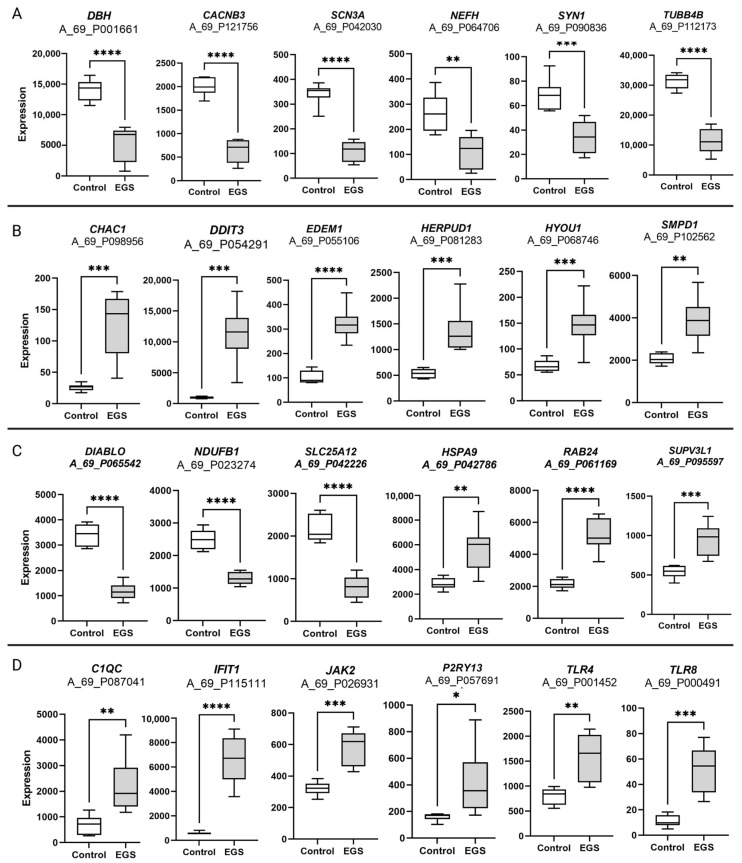
Expression of up- and downregulated genes in EGS. Box and whisker plots for expression levels of selected genes in control (white boxes) and EGS (gray boxes) CCG samples. Horizontal bar shows median; box shows 1st to 3rd quartile, and vertical bars show maximum and minimum values. Y-axis shows the normalized intensity values (expression level). * 0.01 < *p* ≤ 0.05; ** 0.001 < *p* ≤ 0.01; *** 0.0001 < *p* ≤ 0.001; **** *p* ≤ 0.0001. (**A**) Representative genes that were reduced in EGS samples (from cluster002). (**B**) Representative genes that were increased in EGS samples (from cluster009 and cluster010). (**C**) Representative nuclear genes encoding mitochondrial proteins. (**D**) Representative macrophage/microglia genes.

A number of transcript clusters showed high expression in control animals and lower expression in EGS. There were 1579 probes in these downregulated clusters, comprising 804 unique annotated genes and 346 probesets with no annotation. The largest downregulated cluster was cluster002 (Figure 1D). Transcripts in cluster002 represented genes associated with the nervous system, such as those encoding sodium, potassium and calcium ion channels or proteins involved in synaptic transmission (summarized in Table 2; full list available in Table S2). Functional annotation clustering with DAVID of the full set of differentially under-expressed genes in EGS showed high enrichment for neurological-related KEGG pathways such as dopaminergic synapse (FDR 7.2 × 10^−4^) and amphetamine addiction (FDR 2.4 × 10^−4^) as well as Parkinson’s, prion, Alzheimer’s and Huntington’s diseases (FDR 5.0 × 10^−8^, 2.0 × 10^−5^,1.1 × 10^−5^ 2.4 × 10^−4^ respectively). There were also many genes associated with ion channels (for example, with the keywords potassium ion transport, voltage-gated channel and inorganic ion transmembrane transport; FDR 2.8 × 10^−3^, 1.1 × 10^−4^ and 1.6 × 10^−2^, respectively). There was apparent enrichment for terms related to microtubule structure, including microtubule cytoskeleton organization (FDR 1.3 × 10^−3^) and various tubulin-related terms, in the genes that were higher in controls. Examples of transcripts that were reduced in EGS are shown in Figure 2A and a summary of the genes and functions is presented in Table 2.

### 3.6. The Stress Response Was Upregulated in EGS

Clusters with higher average gene expression in EGS samples than in controls contained 1352 probes (representing 671 unique genes and 236 unannotated probes). There was greater variability in expression levels in these genes for the EGS horses than for the control horses, as can be seen in the examples shown in Figure 2B. The largest clusters with an average expression that was higher in EGS samples were cluster009 and cluster010. These contained genes that tend to be expressed in situations of stress and extensive cell death. For example, *CHAC1* and *DDIT3* (both encoding proteins involved in the unfolded protein response) were found in cluster009. Other genes encoding stress response proteins were also upregulated in EGS (examples in Table 2; full list available in Table S2). DAVID analysis showed that GO terms and pathways associated with apoptosis, the stress response and the endoplasmic reticulum were over-represented (for example, regulation of programmed cell death: FDR 1.3 × 10^−6^; response to stress: FDR 6.7 × 10^−6^; response to unfolded protein: FDR 7.0 × 10^−4^; endoplasmic reticulum unfolded protein response: FDR 2.5 × 10^−3^). Terms and pathways related to protein synthesis and modification were also enriched in this group of upregulated genes, for example, KEGG pathways protein processing in the endoplasmic reticulum (FDR 9.9 × 10^−5^), ribosome biogenesis (FDR 8.3 × 10^−10^), and positive regulation of the protein modification process (FDR 7.2 × 10^−2^). Table 2 contains a summary of the genes and functions found in cluster009 and cluster010.

### 3.7. Mitochondrial Function Was Impacted in EGS

Mitochondrial function appeared severely disrupted in the EGS horses (Table 3). Many genes encoding proteins of oxidative phosphorylation (complexes I, II, IV and V) were downregulated in EGS, as well as genes reflecting other mitochondrial functions, such as protein targeting and import, small-molecule transport and formation of mitochondria. DAVID analysis showed that the GO term mitochondrion and the KEGG pathway oxidative phosphorylation were significantly enriched in the downregulated cluster002 (FDR 1.5 × 10^−1^ and 9.4 × 10^−3^, respectively). *DIABLO* (4 probes; 2.8-fold higher in controls), which encodes a pro-apoptotic cytoplasmic protein and also has a role within the mitochondrion in the regulation of phospholipid biosynthesis, was the most differentially expressed downregulated mitochondrial protein gene. *SLC25A12* (1 of 2 probes; 2.7-fold higher in controls; cluster002), encoding an aspartate/glutamate antiporter of the inner mitochondrial membrane, was also significantly depleted in EGS. In contrast, genes related to mitochondrial dysfunction and response to stress were in the large clusters of genes upregulated in EGS (cluster009 and cluster010). For example, RAB24 (*RAB24* gene in cluster010) is associated with autophagy-related processes, the mitochondrial ATP-dependent RNA helicase SUPV3L1 (*SUPV3L1* gene in cluster009) may be involved in protection from apoptosis and HSPA9 (*HSPA9* gene in cluster009) is a heat-shock (molecular chaperone) protein of the mitochondrial membrane. The results for representative nuclear genes encoding mitochondrial proteins are shown in Figure 2C and the expression of mitochondrial protein genes in all samples is presented in Table S3.

### 3.8. Markers of Inflammation Were Seen in EGS

The GSEA analysis indicated that genes associated with inflammation were upregulated in EGS, and GO terms associated with inflammation and immunity were enriched in the list of genes with increased expression in EGS. In particular, cluster013 (Figure 1D) contained many markers of inflammation. GO terms and pathways enriched in this cluster included immune response (FDR 1.6 × 10^−8^), inflammatory response (FDR 9.3 × 10^−5^), antimicrobial humoral immune response mediated by antimicrobial peptides (FDR 6.9 × 10^−6^), defense response (FDR 4.6 × 10^−4^), the chemokine signaling pathway (FDR 3.2 × 10^−6^) and the TNF signaling pathway (FDR 4.8 × 10^−9^). All the members of the IFN-induced protein with tetratricopeptide repeats (IFIT) family in the analysis (*IFIT1*, *IFIT1B*, *IFIT3*, *IFIT5*) were found in the clusters with higher expression in EGS horses. In particular, the *IFIT1* gene (also known as *ISG56*) was increased 11.1-fold in EGS-affected animals (Figure 2D). Other transcripts associated with inflammation included those encoding toll-like receptors (notably *TLR2*, *TLR3*, *TLR4* and *TLR8*), which respond to bacterial and viral proteins and/or DNA, and *JAK2*, encoding a component of the interferon signaling cascade. The *SAA1* gene transcript, encoding serum amyloid A1 which is an acute-phase protein that is involved in response to inflammation and tissue injury, was also higher in some EGS horses. Some other markers of microglial/macrophage activation were also increased in EGS CCG samples. These included genes encoding the three subunits of complement factor 1 (*C1QA*, *C1QB* and *C1QC*) and the genes encoding a purinergic receptor (*P2RY13*), a macrophage transcription factor (*SPI1* gene) and the macrophage mannose receptor (*MRC1* gene). The marker of the microglia neurodegenerative phenotype, *CLEC7A*, was marginally increased, with one EGS sample being 10-fold higher than the controls. However, other macrophage/microglia genes were unchanged (*P2RY12*, *TREM2*, *CTSS*) or downregulated (*TMEM119*, *APOE*) in EGS samples. Representative inflammatory response genes are shown in Figure 2D, where the large variation in expression levels in the EGS horses can be seen. These results indicate that EGS horses are undergoing an inflammatory response in the CCG.

### 3.9. IPA of Gene Clusters Showed the Impact of EGS on Mitochondria

To identify specific functional pathways and/or processes which may be perturbed in EGS, the 485 upregulated and 721 downregulated genes identified through network clustering analysis with BioLayout were submitted to Ingenuity Pathway Analysis (IPA) software (summarized in Appendix A Table A1). Many of the identified genes were associated with a range of neurological disorders, suggesting conservation of molecular processes between acute/sub-acute EGS and other neurodegenerative conditions. The majority of alterations identified here were associated with downregulation of mitochondrial function and upregulation of the unfolded protein response, including altered ubiquitination, previously reported at the protein level [14] (Table S4). The majority affected the function of mitochondrial inner membrane complexes I, III, IV and V, involved in oxidative phosphorylation (Table S3). Analysis of the IPA annotations associated with the nervous system, transport and processing of molecules and metabolism is therefore consistent with the impressions gained from initial analysis of gene expression patterns and the analysis of GO terms.

### 3.10. Proteomic Analysis of EGS and Control Samples

The earlier proteomic analysis [14] was compromised by the previously limited annotation of the horse proteome. We therefore processed the raw data again using the more recent EquCab3.0 (updated January 2022). This resulted in an almost 2-fold increase in the annotated proteins (2311 for the previous paper; 4125 in the present study) and hence provided considerably more information about the proteome in EGS. Filtering on a fold change of at least 1.2 in either direction, we found 283 annotated proteins that were reduced in EGS and 336 that were increased. The encoded proteins of twenty-seven of the genes in microarray cluster002 (reduced in EGS) also showed a negative fold change in the protein analysis. These included TUBB4A (β-tubulin class IVA), NDUFB3 (NADH: Ubiquinone Oxidoreductase Subunit B3) and the neurofilament proteins NEFH and NEFM. In addition, there were 32 proteins encoded by genes that had increased expression in EGS that showed a positive fold change in the protein analysis, including the somatic cytochrome C (CYCS) and the heat-shock proteins HSPA5 and HSPA9. Several gene/protein pairs showed changes in opposite directions. For example, dopamine β-hydroxylase protein was nearly two times higher in EGS than controls but *DBH* mRNA was reduced in EGS. This may reflect the different samples, a longer half-life of the protein than the mRNA, or a feedback regulation mechanism that reduced transcription of the *DBH* gene in response to the disease.

**Figure 3:**
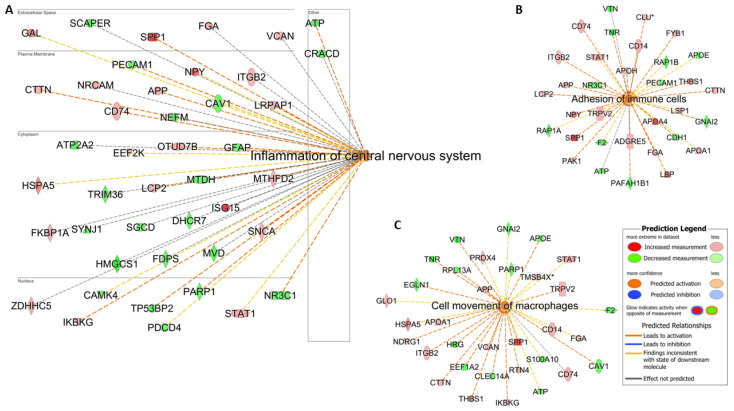
Networks identified by pathway analysis show inflammation in the central nervous system. Biological processes and regulators are color-coded by predicted activation state: orange for activation and blue for inhibition, with darker shades indicating higher confidence scores. Edges represent relationships: orange indicates activation of the downstream node, blue indicates inhibition, and yellow highlights findings inconsistent with the downstream state. Pointed arrowheads denote predicted activation of the downstream node, while blunt arrowheads denote predicted inhibition. Molecules in green are downregulated and those in red are upregulated; an asterisk (*) indicates that multiple identifiers map to the same molecule. Analyses were performed using Ingenuity Pathway Analysis software (v24.0.1). (**A**) Inflammation of central nervous system. (**B**) Adhesion of immune cells. (**C**) Cell movement of macrophages.

Similar to the transcriptomic data, proteomic analysis using IPA (summarized in Table A2) revealed downregulation of several neurological function pathways, including NCAM signaling for neurite outgrowth, Neurexin–neuroligin signaling, Agrin interactions at the neuromuscular junction, Reelin signaling in neurons, and the Endocannabinoid developing neuron pathway (Table S5). Moreover, pathways associated with inflammatory responses were upregulated in EGS horses, mirroring the transcriptomic analysis and highlighting inflammation as a convergent feature at both the gene and protein level (Figure 3). Several proteins associated with the activated Cellular Response to Heat Stress pathway were also differentially expressed in EGS cases, including AKT1S1, ATP, EEF1A1, HSPA1A, HSPA2, HSPA5, HSPA9, HSPB8, HSPH1, NUP62, POM121C, and SERPINH. Finally, mitochondrial dysfunction observed in EGS mRNA was also reflected in the proteomic dataset. In particular, IPA revealed activation of the Mitochondrial Protein Degradation canonical pathway, which included the following differentially expressed proteins: ALDH18A1, APP, ARG2, ATP5A1, ATP5PF, COX5A, COX5B, HSPA9, HSPD1, MT-CO2, SHMT2, SSBP1 and TIMM9.

To gain a deeper understanding of the proteomic data, functional analysis was also conducted using DAVID annotation software. Consistent with the IPA, DAVID results identified significant enrichment in pathways and biological processes associated with nervous system pathology, inflammatory response, metabolic processes, mitochondrial dysfunction, and stress response. In line with the transcriptomic data, KEGG pathway analysis of the upregulated proteins highlighted a strong enrichment of neurodegenerative disease pathways, including Parkinson’s disease, Alzheimer’s disease, prion disease, amyotrophic lateral sclerosis, and Huntington’s disease, alongside general pathways of neurodegeneration. Comprehensive biological process and KEGG pathway enrichment analyses are available in Table S6.

**Figure 4:**
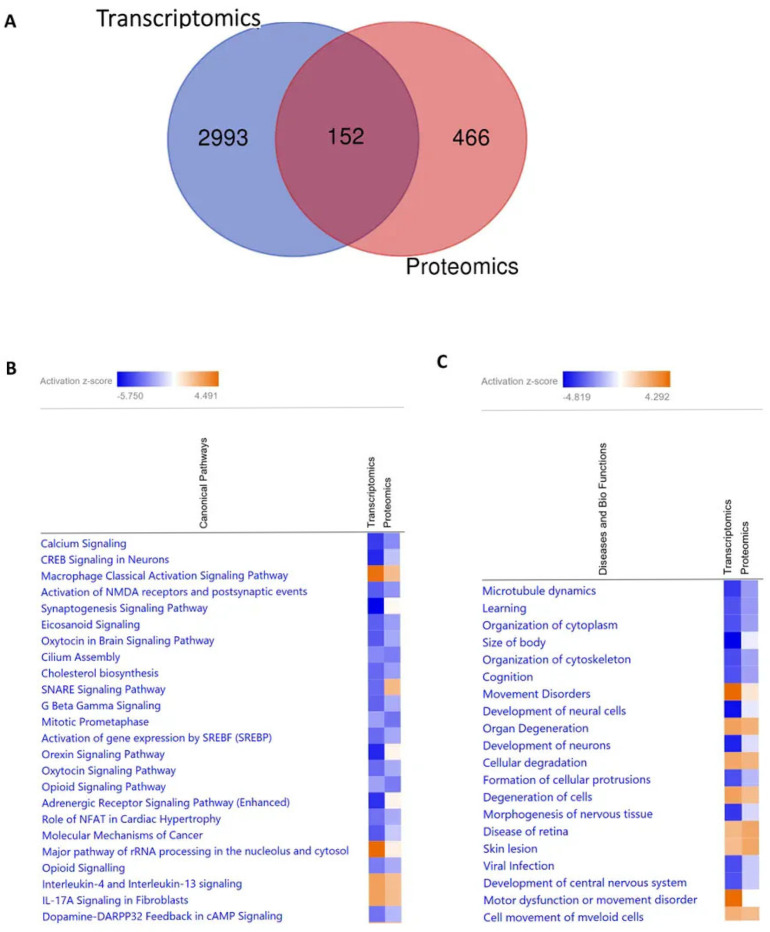
Multi-omics analysis reveals distinct transcriptomic and proteomic signatures with pathway-specific perturbations. (**A**) Venn diagram showing the overlap between differentially expressed molecules identified through transcriptomics (blue circle, *n* = 3145 differentially expressed unique genes) and proteomics (red circle, *n* = 618 differentially expressed unique proteins) analyses. Numbers indicate unique molecules in each dataset and the overlap between approaches (*n* = 152). (**B**) Ingenuity Pathway Analysis (IPA) canonical pathway heatmap showing activation z-scores for significantly enriched pathways across transcriptomics and proteomics datasets. Pathways are ranked by significance and colored according to predicted activation state: blue indicates pathway inhibition, orange indicates pathway activation, and white represents no significant activation prediction. z-scores range from −5.750 (maximum inhibition) to +4.491 (maximum activation). (**C**) IPA disease and bio-function analysis showing activation z-scores for biological processes and disease-associated functions. Color scheme as in panel B, with z-scores ranging from −4.819 to +4.292. Functions are categorized and displayed based on their predicted activation or inhibition status across the integrated multi-omics dataset.

Interestingly, despite the limited overlap between the transcriptomic and proteomic datasets (4.2%) (Figure 4A), a remarkable number of pathways identified as enriched by IPA and DAVID were shared between the two datasets. IPA comparative analysis also revealed substantial overlap of canonical pathways and diseases and bio-functions between the transcriptomic and proteomic datasets (Figure 4B,C).

IPA of the integrated datasets (Figure 4B,C) revealed significant perturbations in multiple biological processes. Canonical pathway analysis (Figure 4B) showed prominent activation of inflammatory signaling pathways including calcium signaling, CREB signaling in neurons, and macrophage activation, alongside inhibition of metabolic pathways such as cholesterol biosynthesis and various neurotransmitter signaling cascades. Disease and bio-function analysis (Figure 4C) indicated activation of processes related to cellular organization and movement, while pathways associated with neurological functions indicated mainly inactivation of nervous system development and function.

## 4. Discussion

This study represents the first analysis of the transcriptome of CCG in horses, conducted in parallel with proteomic profiling. A gene expression microarray was used to examine gene expression of the CCG in EGS-affected and control horses. The datasets were then analyzed using network analysis to allow identification of clusters of genes that were co-expressed and either up- or downregulated in the CCG of horses with EGS. Molecular analyses are frequently performed on experimentally or bioinformatically pooled samples. For example, the proteomic dataset consisted of two technical replicates of a sample pooled from six horses [14]. Similarly, the GSEA creates a weighted average expression value for each gene and accounts statistically for variation in expression of that gene among samples. These approaches minimize “noise” generated by differences in age, sex, breed, environment, etc., but lose information that may be gained by examining individual values. In this study we utilized the power of network analysis to identify clusters of co-expressed genes across individual samples. This highlighted the inter-individual variability but also identified sets of genes with altered expression in all cases, such as cluster013. The inter-individual variation in transcriptomic profiles likely reflects a combination of genetic diversity, environmental influences, and differences in disease stage or severity among horses, consistent with our previous transcriptomic studies of other outbred animals including horses [30] and humans [31]. Such variability highlights the need to account for individual biological differences when interpreting gene expression data.

Initially we observed that many of the genes downregulated in EGS-affected CCG samples were linked with the nervous system, including synapse-related, proton pump-related and ion-channel genes (Table 2). The reduced expression of neurological genes in the CCG samples of EGS horses was confirmed by the subsequent DAVID analysis and IPA which found genes associated with the term neurological disease were downregulated in EGS (Tables S4 and S5). This is consistent with the damage and loss of neurons seen microscopically in CCG samples from EGS horses and suggests that by the time of death, the CCG had been severely affected. A similar result was found in a study of the cervical spinal cord of horses affected with vitamin E-deficient neuroaxonal dystrophy [32]. Many genes involved in ATP metabolism were downregulated. One of these, *ATP13A2*, is mutated in a form of juvenile Parkinson’s syndrome in humans associated with iron deposition and heavy-metal toxicity in the brain [33,34], consistent with neurological abnormalities in EGS horses. The majority of genes upregulated in EGS were associated with cell stress and death, including the unfolded protein response, again consistent with the histological changes. This is supported by our expanded proteomic study of a separate set of EGS horses that showed dysregulation of proteins indicative of cellular stress, including protein misfolding/aggregation responses. At least two upregulated genes, *NEDD4* and *HYOU1*, are associated with hypoxia and oxidative stress. Over-expression of *HYOU1* prevents endoplasmic reticulum stress in mice [35], while increased levels of NEDD4 protein have been found in neurodegenerating tissues [36]. In addition, the upregulated transcription factor ATF6 is an activator of the endoplasmic reticulum stress response [37]. These results indicate that the neurons in EGS were undergoing cellular stress. There was little evidence of proliferation or DNA replication in the CCG from diseased horses and two genes involved in suppression of the cell cycle were upregulated, although the MYC gene, encoding a transcription factor which is activated by mitogenic signals and can drive cell proliferation, was also upregulated [38]. Since MYC also controls apoptosis and the gene encoding TFAP2B, a suppressor of MYC-induced apoptosis, was downregulated, it is likely that excess MYC is associated with the cell death seen in the EGS samples rather than cell proliferation. IPA showed that translation of mRNA was associated with five upregulated genes and metabolism of protein was associated with 12 upregulated genes, primarily initiation factor genes, although there was no evidence of increased expression of ribosomal protein genes, suggesting that coordinated regulation of the protein synthetic machinery has failed in the EGS horses.

Mitochondrial function was clearly disrupted in EGS. This is consistent with observations of abnormality in mitochondrial DNA in human neurodegenerative conditions [39,40,41] and in animal models [42]. EGS CCG neurons have been reported to have more mitochondria than control neurons, with similar morphologies, although more slender in shape (reviewed in [43]). The reduction in mRNA for mitochondrial proteins and increase in proteins involved in mitochondrial damage repair suggests that the observed mitochondria are likely to be deficient in oxidative phosphorylation and the increase in numbers may be a cellular response to this deficiency.

Immune activation appeared to be a factor in EGS, although the level of gene expression varied considerably among both control and EGS horses (as can be seen in Figure 2D). This may reflect the exposures of the control and EGS horses to a range of pathogens. Although there was no histological evidence of macrophage infiltration into the ganglia of affected horses (Figure 1A), there was evidence of activation of macrophages/microglia, for example, increased expression of genes for the interferon-inducible IFIT family of proteins and the toll-like receptor (TLR) family. IFIT proteins bind RNA and are key players in antiviral responses [44,45,46]. They are highly expressed by activated macrophages (see http://biogps.org [47]). TLRs are pattern-recognition receptors that sense infectious organisms and initiate a signaling cascade aimed at eliminating the infected cell and the pathogen [48]. They are highly expressed in myeloid cells (http://biogps.org). TLR activation results in the induction of cytokines such as interleukins and interferons. The interferon genes, those encoding interferon receptors and those for the STAT transcription factors were not generally upregulated and tended to show a horse-specific rather than disease-specific pattern. Only *JAK2* from the interferon signaling pathway was consistently upregulated in EGS. Other interferon-inducible genes, such as *PRKRA*, and other genes involved in immune responses such as *MAVS*, were downregulated. IFIT proteins have been linked with cell death via apoptosis [49], and it is possible that their role in EGS is related to the elimination of damaged neurons, in line with an increase in SAA1 in response to tissue damage. These results are consistent with a microglial neurodegenerative phenotype in the EGS CCGs.

The presence of fibrosis in EGS CCG samples is an inconsistent histological finding [1]. IPA showed differential protein levels for canonical pathways relating to the extracellular matrix, collagen assembly and fibrosis. In the transcriptomic data there were examples of mesenchymal markers upregulated in EGS CCGs, particularly noticeable in EGS1 and EGS6. For example, expression of sphingomyelin phosphodiesterase 1 (*SMPD1* gene), also known as ASMASE or lysosomal acid SMASE, was consistently increased in EGS (2.79-fold). This protein is involved in sphingomyelin-to-ceramide production at an acid pH, and ceramide has been implicated in activation of cell death signaling pathways (reviewed in [50]). It shows high expression in some mesenchymal and neuronal cell types in mice and humans. The similar gene *SMPD2* (encoding sphingomyelin phosphodiesterase 2, also known as NSMASE or neutral sphingomyelinase) was downregulated. This gene is required for neuronal viability mediated through neurotrophins [51]. Thus, the opposite functions of these proteins are reflected in their differential expression in EGS and control horses. Another pair of differentially regulated genes consistent with the disruption of connective tissue in EGS CCGs was *ESYT1* (downregulated in EGS) and *ESYT2* (upregulated in EGS). These genes encode extended synaptotagmin 1 and 2. *ESYT1* is strongly expressed in lymphoid tissues, macrophages and mast cells, but also found in dorsal root ganglia and the spinal cord, suggesting that it is expressed by immune-associated cells in the CCG. *ESYT2* is also expressed in mast cells, but has high expression in osteoblasts and other mesenchymal lineage cells. Expression of this gene and its paralogue *ESYT3* is important in cell survival under stress [52] and upregulation in EGS is consistent with increased levels of stress response genes.

The network clustering approach identifies co-expressed genes. These are likely to be coregulated and to have similar functions [53,54,55]. All the clusters of increased or decreased transcript expression contained unannotated probes, representing novel transcripts. Although little is known about the genes that produce these transcripts, their presence in a cluster of known genes provides functional annotation. For example, the clusters of genes that were lower in EGS contained 346 unannotated probes and 24 genes with only an LOC gene name, which are likely to have similar functions to the known transcripts in these clusters, many of which encode neuronal and mitochondrial proteins. Similarly, there were 236 unannotated probes and 48 LOC genes in the clusters that were increased in EGS, which may be involved in stress responses. These unannotated genes should be characterized and may reveal proteins of significance in human neurodegeneration.

Proteomic pathway analyses revealed downregulation of key neuronal signaling pathways, alongside upregulation of inflammatory responses, stress-related proteins, and mitochondrial dysfunction, closely paralleling the transcriptomic findings. Even though the overlap between the transcriptomic and proteomic datasets was limited, this was not unexpected, as discrepancies between gene and protein expression are well documented [56,57] and may arise from factors such as post-transcriptional regulation, differences in molecular stability and degradation, the different samples used or sampling variation between individuals [58]. In addition, only 4125 proteins were annotated, in contrast to more than 32,000 genes, limiting the possibilities for overlap. Nevertheless, functional analyses showed strong parallels between the two datasets, consistent with our previous observations [59,60] highlighting strong convergence on inflammation, neurodegeneration, metabolic disruption, and impaired nervous system function as central features of EGS.

One limitation of the study is that the EGS samples in both the transcriptomic and proteomic analyses were from horses with advanced neurodegeneration, despite the short clinical course. Indeed, the median times from onset of the first abnormal clinical sign to sample collection were only 1.5 and 2 days for acute and sub-acute cases, respectively. Obtaining samples from earlier stages of EGS is impractical. Our results thus show changes in the transcriptome and proteome of horses with advanced neurodegeneration that may not reflect causative or progressive changes as the disease evolves.

A second limitation is the small sample size, which resulted in a wide spread of ages. Although there was no indication of an age or sex effect (Figure 1C), we noted that EGS1 frequently had the largest deviation in expression level from the control samples. This animal was both the youngest of the subjects and the only intact male in the sample, and our results suggest that this horse had a more extreme response to the disease. However, the proteomics analysis, using a different set of horses, gave similar results to the transcriptomic analysis, supporting the overall findings and suggesting that the perturbations in neuronal function, mitochondrial activity, stress response and inflammation markers are a real consequence of the disease.

Overall, biobank samples could play a key role in solving equine grass sickness, an emerging priority, and the current manuscript, presenting multi-omic analyses of equine CCG from EGS cases, contributes toward this goal. This collaborative, multidisciplinary approach will be key to uncovering disease pathogenesis and the toxin transmission route to advance prevention and treatment for this devastating condition [61,62].

## 5. Conclusions

In summary, the transcriptome and proteome of the CCG in horses with acute or sub-acute EGS reflected the substantial cell death found at the time of necropsy. Gene expression patterns were consistent with the loss of neurons, mitochondrial dysfunction and upregulation of a range of stress-associated genes, presumably in an attempt by affected cells to restore homeostasis. Inflammatory processes including a possible microglial neurodegenerative phenotype were suggested by the gene expression changes. The striking variability in gene expression of both EGS and control horses highlights inter-individual differences, which can confound analysis where pooled results are used. Examining mRNA levels using network clustering allowed us to identify up and downregulated clusters of genes in spite of these individual differences. While the detection of an mRNA does not mean that the protein is also present, it is a marker of the genetic response of the cell to the insult causing EGS and hence provides an indication of the potential processes that cells can adopt in this situation, supported by the more limited proteomic data.

## Figures and Tables

**Table 1:** Details of horses included in the transcriptomic study.

Sample	Age (Years)	Sex ^a^	Breed ^b^	Diagnosis ^c^	Duration of Clinical Signs of EGS Prior to Sampling (Days) ^d^
Control 1	6	F	TB cross	Laminitis	N/A
Control 2	9	MN	Arab cross	Undifferentiated chronic weight loss; EGS ruled out	N/A
Control 3	11	MN	TB cross	Chronic lameness	N/A
Control 4	12	F	ID cross	Pedal bone fracture	N/A
Control 5	15	MN	ID cross	Laminitis	N/A
Control 6	35	MN	Welsh Sec C	Mandibular neoplasm	N/A
EGS 1	0.5	M	Cob	Sub-acute EGS	2
EGS 2	3	F	Selle Français	Sub-acute EGS	2
EGS 3	6	MN	Warmblood	Acute EGS	2
EGS 4	6	MN	Warmblood	Acute EGS	1
EGS 5	7	F	Highland	Acute EGS	2
EGS 6	7	MN	ID cross TB	Sub-acute EGS	5
EGS 7	9	F	Polo pony	Sub-acute EGS	2
EGS 8	20	F	TB cross	Acute EGS	1

^a^ M—whole male; MN—neutered male; F—female. ^b^ ID—Irish Draft; TB—Thoroughbred. ^c^ All grass sickness diagnoses were histologically confirmed. EGS—equine grass sickness. ^d^ All ganglia samples were collected within two hours of euthanasia, except for control 6 where the tissue was collected within 3.5 h after euthanasia and EGS4 where the tissue was collected within 10 h.

**Table 2:** Examples of down- and upregulated genes from the largest clusters.

Functional Grouping	Downregulated in EGS (from Cluster002)	Upregulated in EGS (from Cluster009 and Cluster010)
Synapse	*ADRA1A*, *AGRN*, *AMPH*, *CNTN1*, *CNTN5*, *CNTN6*, *ESYT1*, *NXPH1*, *SLC18A1*, *SYN2*, *SYNGR3*, *SYT3*, *VAMP1*	*ESYT2*, *SEZ6L*
Neurotransmission	*DBH*, *DDC*, *GABRR2*, *GATA3*, *GRIA2*, *GRM7*	
Ion channel	*CACNA2D3*, *CACNB3*, *CACNG2*, *CLCN4*, *KCNC2*, *KCND1*, *KCNIP4*, *KCNK10*, *KCNMA1*, *KCNQ5*, *KCNS3*, *SCN2A*, *SCN3A*, *SCN9A*	
ATP metabolism	*ATP1A1*, *ATP1A3*, *ATP5H*, *ATP6V1G2*, *ATP8A1*, *ATPIF1*	*ATP11B*, *ATP6V0A2*
Ubiquitin	*NEDD8*, *UBE2E3*, *UBE2T*, *USP20*	*HACE1*, *NEDD4*, *NUB1*, *UBA5*, *UBE2D3*, *UCHL3*, *USP15*
Proteasome components		*PSMA7*, *PSMB9*, *PSME4*
Endoplasmic reticulum		*EDEM1*, *TFG*
Immune system	*FAM129C*, *IFITM10*, *IL5*, *IRF6*, *MAVS*, MIF, *PRKRA*, *PRRC2A*	*BTG3*, *IFIT1*, *IFIT1B*, *IFRD1*, *ISG15*, *TLR4*
Stress responses	*HSPB6*, *STIP1*	*ATF6*, *CHAC1*, *DDIT3*, *HERPUD1*, *HSPA4*, *HSPA5*, *HSPA9*, *HYOU1*, *NEDD4*, *XBP1*
Regulation of transcription	*IRF6*, *MYT1L*, *TFAP2B*, *TFAP2D*, *POLDIP2*, *POLR2J*	*ATF6*, *BTF3*, *GTF2H1*, *LOC100069633*, *POLR1C*, *POLR3D*, *TAF12*
Cell division	*CEND1*, *DNAJC12*, *GAS2*, *INCENP*, *MCM3*, *NUF2*, *PMS2*, *RMND1*, *URGCP*	*BUB1*, *DNAJA3*, *DNAJB9*, *DNAJC2*, *DNAJC3*, *GSPT*, *MCM8*, *ORC4*, *TOP1*

**Table 3:** Expression of genes encoding mitochondrial proteins in EGS.

Functional Grouping	Downregulated in EGS (from Cluster002)	Upregulated in EGS (from Cluster009 and Cluster010)
Oxidative phosphorylation complex I	*NDUFA3*, *NDUFA8*, *NDUFA9*, *NDUFA10*, *NDUFA13*, *NDUFAB1*, *NDUFB1*, *NDUFB3*, *NDUFS3*	
Oxidative phosphorylation complex III	*UQCRH*	
Oxidative phosphorylation complex IV	*COX6A1*	
Oxidative phosphorylation complex V	*ATP5H*	
Mitochondrial transcription and translation	*IARS2*, *MRPL28*, *MRPS6*, *MRPS17*	*MRPS10*, *MRPS16*, *MRPS30*, *MRPS31*, *MRPS35*
Apoptosis-associated	*DIABLO*	*SOD2*, *BID*
Other mitochondrial protein genes	*COX18*, *FIS1*, *MIPEP*, *IMMP1L*, *TOMM6*, *TOMM7*, *SLC25A1*, *SLC25A4*, *SLC25A12*, *SLC25A14* *IVD*, *MAOA*, *MAOB*, *MAVS*	*HSPD1*, *IMMP2L*, *TOMM20*, *SLC25A32*, *SLC25A5*

## Data Availability

The original contributions presented in this study are included in the article/Supplementary Materials. Further inquiries can be directed to the corresponding authors.

## Supplementary Materials

The following supporting information can be downloaded at https://www.mdpi.com/article/10.3390/cells15151328/s1. Table S1: GSEA analysis of transcriptomic data. Table S2: List of network clusters generated by BioLayout analysis and average expression profiles. Table S3: Expression of genes encoding mitochondrial proteins in all samples. Table S4: Ingenuity Pathway Analysis of transcriptomic data. Table S5: Ingenuity Pathway Analysis of proteomic data. Table S6: DAVID analysis of the largest up and down-regulated clusters.

## Abbreviations

The following abbreviations are used in this manuscript:

CCG	Cranial (superior) cervical ganglion
DAVID	Database for Annotation, Visualization and Integrated Discovery (https://davidbioinformatics.nih.gov/, accessed 31 May 2026)
EGS	Equine grass sickness, also known as equine dysautonomia
F	Female
FDR	False-discovery rate
GSEA	Gene-set enrichment analysis (https://www.gsea-msigdb.org/gsea/index.jsp, accessed 31 May 2026)
IPA	Ingenuity pathway analysis (https://digitalinsights.qiagen.com, accessed 31 May 2026)
KEGG	Kyoto Encyclopedia of Genes and Genomes (https://www.genome.jp/kegg/pathway.html, accessed 31 May 2026)
M	Male
MCL	Markov clustering algorithm
MN	Neutered male
nPLA2	Neurotoxic phospholipase A2

## Appendix A

**Table A1:** Ingenuity Pathway Analysis of expression of up- and downregulated genes in EGS.

Category	Function Annotation	Molecules	*p*-Value	Number of Molecules
Disease/Disorder	movement disorder	Increased in EGS: *CANX*, *TAC1*	1.44 × 10^−3^	21
		Decreased in EGS: *BASP1*, *CACNA2D3*, *CACNB3*, *CACNG2*, *CAMK2B*, *CAMK4*, *CHRNB4*, *DLG2*, *ELAVL2*, *GNAO1*, *IP6K2*, *KCNC2*, *KCNMA1*, *NDUFA13*, *PPP3CB*, *SCN2A*, *SERPINI1*, *SYN1*, *VSNL1*		
	neuromuscular disease	Increased in EGS: *TAC1*	4.08 × 10^−3^	18
		Decreased in EGS: *BASP1*, *CACNA2D3*, *CACNB3*, *CAMK2B*, *CAMK4*, *DBC1*, *DLG2*, *ELAVL2*, *GNAO1*, *IP6K2*, *KCNMA1*, *NDUFA13*, *PPP3CB*, *SCN2A*, *SERPINI1*, *SYN1*, *VSNL1*		
	behavior	Increased in EGS: *CANX*, *SIRT1*, *TAC1*	1.33 × 10^−2^	16
		Decreased in EGS: *AMPH*, *APBB1*, *APC*, *CACNB3*, *CACNG2*, *CAMK4*, *CSPG5*, *DNM1*, *GNAO1*, *KCNC2*, *KCNMA1*, *SERPINI1*, *WASF1*		
	dyskinesia	Increased in EGS: *TAC1*	2.26 × 10^−3^	15
		Decreased in EGS: *BASP1*, *CACNA2D3*, *CACNB3*, *CAMK2B*, *CAMK4*, *CHRNB4*, *ELAVL2*, *GNAO1*, *IP6K2*, *KCNMA1*, *NDUFA13*, *SCN2A*, *SERPINI1*, *VSNL1*		
	disorder of basal ganglia	Increased in EGS: *TAC1*	1.37 × 10^−2^	15
		Decreased in EGS: *BASP1*, *CACNA2D3*, *CACNB3*, *CAMK2B*, *CAMK4*, *DLG2*, *ELAVL2*, *GNAO1*, *IP6K2*, *NDUFA13*, *SCN2A*, *SERPINI1*, *SYN1*, *VSNL1*		
	Huntington’s disease	Increased in EGS: *TAC1*	1.00 × 10^−2^	13
		Decreased in EGS: *BASP1*, *CACNA2D3*, *CACNB3*, *CAMK2B*, *CAMK4*, *ELAVL2*, *GNAO1*, *IP6K2*, *NDUFA13*, *SCN2A*, *SERPINI1*, *VSNL1*		
	schizophrenia	Increased in EGS: *SIRT1*, *TAC1*	7.59 × 10^−3^	11
		Decreased in EGS: *AMPH*, *APC*, *CACNG2*, *CHRNB4*, *DLG2*, *GRINA*, *PPP3CB*, *SERPINI1*, *VSNL1*		
	seizures	Increased in EGS: *TAC1*	4.71 × 10^−3^	8
		Decreased in EGS: *AMPH*, *CACNA2D3*, *CHRNB4*, *GNAO1*, *KCNC2*, *SCN2A*, *SYN1*		
	epilepsy	Increased in EGS: *TAC1*	1.24 × 10^−4^	7
		Decreased in EGS: *CACNA2D3*, *KCNC2*, *KCNMA1*, *SCN2A*, *SPTAN1*, *SYN1*		
	ataxia	Increased in EGS: *CANX*	5.24 × 10^−3^	6
		Decreased in EGS: *CACNG2*, *CAMK2B*, *CAMK4*, *KCNMA1*, *SCN2A*		
Function/Process	transport of molecules	Increased in EGS: *BET1*, *GRB10*, *SELS*, *SIRT1*, *TAC1*	1.29 × 10^−3^	22
		Decreased in EGS: *AP2A1*, *CACNA2D3*, *CACNB3*, *CACNG2*, *CAMK2B*, *CAMK2G*, *CAMK4*, *CHRNB4*, *DPP6*, *IP6K2*, *KCNMA1*, *RYR2*, *SCN2A*, *SLC22A4*, *SLC39A7*, *SYN1*, *USO1*		
	metabolism of proteins	Increased in EGS: *CANX*, *EDEM1*, *EIF1*, *EIF2S2*, *EIF3A*, *EIF3C/EIF3CL*, *EIF5*, *GTPBP4*, *H2AFZ*, *SELS*, *SIRT1*, *WARS*	9.55 × 10^−3^	16
		Decreased in EGS: *APC*, *CAPN1*, *MIPEP*, *NDUFA13*		
	neurotransmission	Increased in EGS: None	1.50 × 10^−5^	14
		Decreased in EGS: *AMPH*, *CACNB3*, *CAMK4*, *CHRNB4*, *CSPG5*, *DLG2*, *DNM1*, *DPP6*, *KCNC2*, *KCNMA1*, *NOVA1*, *SCN2A*, *SYN1*, *WASF1*		
	synaptic transmission	Increased in EGS: None	2.05 × 10^−4^	11
		Decreased in EGS: *AMPH*, *CACNB3*, *CAMK4*, *CHRNB4*, *CSPG5*, *DLG2*, *DPP6*, *KCNMA1*, *NOVA1*, *SYN1*, *WASF1*		
	transport of ions	Increased in EGS: None	9.54 × 10^−4^	11
		Decreased in EGS: *CACNA2D3*, *CACNB3*, *CAMK2B*, *CAMK2G*, *CHRNB4*, *DPP6*, *IP6K2*, *KCNMA1*, *RYR2*, *SCN2A*, *SLC22A4*		
	transport of cations	Increased in EGS: None	1.94 × 10^−3^	9
		Decreased in EGS: *CACNA2D3*, *CACNB3*, *CAMK2B*, *CAMK2G*, *DPP6*, *KCNMA1*, *RYR2*, *SCN2A*, *SLC22A4*		
	transport of metals	Increased in EGS: *SLC39A7*	7.86 × 10^−4^	9
		Decreased in EGS: *CACNA2D3*, *CACNB3*, *CAMK2B*, *CAMK2G*, *DPP6*, *KCNMA1*, *RYR2*, *SCN2A*		
	action potential of neurons	Increased in EGS: None	3.89 × 10^−4^	6
		Decreased in EGS: *DLG2*, *KCNC2*, *KCNMA1*, *SCN2A*, *SYN1*, *WASF1*		
	synaptic depression	Increased in EGS: None	6.48 × 10^−4^	6
		Decreased in EGS: *CAMK2B*, *CAMK4*, *CSPG5*, *KCNMA1*, *SYN1*, *WASF1*		
	translation of mRNA	Increased in EGS: *EIF1*, *EIF2S2*, *EIF3C/EIF3CL*, *EIF5*, *WARS*	8.30 × 10^−4^	6
		Decreased in EGS: *NDUFA13*		
	coordination	Increased in EGS: None	1.20 × 10^−2^	6
		Decreased in EGS: *CACNG2*, *CAMK2B*, *CAMK4*, *GNAO1*, *KCNMA1*, *WASF1*		
	transport of Ca^2+^	Increased in EGS: None	4.70 × 10^−3^	5
		Decreased in EGS: *CACNA2D3*, *CACNB3*, *CAMK2B*, *CAMK2G*, *RYR2*		
	I-kappaB kinase cascade	Increased in EGS: *BCL10*, *GOLT1B*, *SIRT1*, *TFG*	1.40 × 10^−2^	5
		Decreased in EGS: *PTPLAD1*		

**Table A2:** Top 20 significant functions related to nervous system development and function identified by IPA from proteomic data, ranked by *p*-value.

Disease or Function Annotation	*p*-Value	z-Score ^a^	Molecules	Total Molecules
**Development of neural cells**	7.76 × 10^−20^	−0.439	ADGRE5, AGRN, AP2A1, APBA1, APBB1, APLP2, APOE, APP, ARHGAP35, ATP, ATP6AP2, BASP1, BSN, CAMK4, CAMSAP2, CAPNS1, CAPRIN1, CAPRIN2, CAST, CAV1, CDH1, CDKL3, CHMP2B, CLSTN1, CLU, COBL, COL4A1, CTTN, DHCR7, DLGAP4, DMD, DPYSL4, EEF1A1, EEF2K, EFHD1, ELMO1, EPB41L3, EPS8, GAP43, GAS7, GFAP, GPRIN1, HEXA, HSPA5, IDH1, IQGAP1, ITGB2, KIF2A, KIF3A, KIF3C, LAMB1, LRPAP1, MAP1B, MARCKS, MECP2, MINK1, MYO6, MYOC, NCAM1, NEFH, NEFL, NEFM, NLGN3, NR3C1, NRCAM, NRDC, PAFAH1B1, PAK1, PEX14, RAB10, RAB6A, RAB6B, RANBP1, RAP1A, RAP1B, RTN3, RTN4, RUFY3, SEZ6L2, SNAP25, SNCA, SNCB, SPAG9, SPARCL1, SPOCK1, STAM, STAU2, SUN1, SV2A, SYT1, THBS1, TNR, TOP2B, UGDH, VAMP2, VGF, YWHAH, ZPR1	98
**Morphogenesis of nervous tissue**	8.11 × 10^−20^	−0.786	AGRN, AP2A1, APBA1, APBB1, APLP2, APOE, APP, ARHGAP35, ATP, ATP6AP2, BASP1, BSN, CAMK4, CAMSAP2, CAPNS1, CAPRIN1, CAPRIN2, CAV1, CDKL3, CHMP2B, CLSTN1, CLU, COBL, CTTN, DMD, DPYSL4, EEF1A1, EEF2K, EFHD1, ELMO1, EPB41L3, EPS8, GAP43, GAS7, GFAP, GPRIN1, HEXA, HSPA5, IQGAP1, KIF2A, KIF3A, KIF3C, LAMB1, LRPAP1, MAP1B, MARCKS, MECP2, MINK1, MYO6, MYOC, NCAM1, NEFH, NEFL, NEFM, NLGN3, NR3C1, NRCAM, NRDC, PAFAH1B1, PAK1, PEX14, RAB10, RAB6A, RAB6B, RANBP1, RAP1A, RAP1B, RTN3, RTN4, RUFY3, SNAP25, SNCA, SNCB, SPAG9, SPOCK1, STAU2, SV2A, SYT1, TNR, TOP2B, VGF, YWHAH, ZPR1	83
**Development of neurons**	1.19 × 10^−19^	−0.653	ADGRE5, AGRN, AP2A1, APBA1, APBB1, APLP2, APOE, APP, ARHGAP35, ATP, BASP1, BSN, CAMK4, CAMSAP2, CAPNS1, CAPRIN1, CAPRIN2, CAST, CAV1, CDH1, CDKL3, CHMP2B, CLSTN1, CLU, COBL, COL4A1, CTTN, DLGAP4, DMD, DPYSL4, EEF1A1, EEF2K, EFHD1, ELMO1, EPB41L3, EPS8, GAP43, GAS7, GFAP, GPRIN1, HEXA, HSPA5, IQGAP1, ITGB2, KIF2A, KIF3A, KIF3C, LAMB1, LRPAP1, MAP1B, MARCKS, MECP2, MINK1, MYO6, MYOC, NCAM1, NEFH, NEFL, NEFM, NLGN3, NR3C1, NRCAM, NRDC, PAFAH1B1, PAK1, PEX14, RAB10, RAB6A, RAB6B, RANBP1, RAP1A, RAP1B, RTN3, RTN4, RUFY3, SEZ6L2, SNAP25, SNCA, SNCB, SPAG9, SPARCL1, SPOCK1, STAM, STAU2, SUN1, SV2A, SYT1, THBS1, TNR, TOP2B, UGDH, VAMP2, VGF, YWHAH, ZPR1	95
**Morphogenesis of neurons**	2.3 × 10^−19^	−0.701	AGRN, AP2A1, APBA1, APBB1, APLP2, APOE, APP, ARHGAP35, ATP, BASP1, BSN, CAMK4, CAMSAP2, CAPNS1, CAPRIN1, CAPRIN2, CAV1, CDKL3, CHMP2B, CLSTN1, CLU, COBL, CTTN, DMD, DPYSL4, EEF1A1, EEF2K, EFHD1, ELMO1, EPB41L3, EPS8, GAP43, GAS7, GFAP, GPRIN1, HEXA, HSPA5, IQGAP1, KIF2A, KIF3A, KIF3C, LAMB1, LRPAP1, MAP1B, MARCKS, MECP2, MINK1, MYO6, MYOC, NCAM1, NEFH, NEFL, NEFM, NLGN3, NR3C1, NRCAM, NRDC, PAFAH1B1, PAK1, PEX14, RAB10, RAB6A, RAB6B, RANBP1, RAP1A, RAP1B, RTN3, RTN4, RUFY3, SNAP25, SNCA, SNCB, SPAG9, SPOCK1, STAU2, SV2A, SYT1, TNR, TOP2B, VGF, YWHAH, ZPR1	82
**Neuritogenesis**	1.32 × 10^−18^	−0.701	AGRN, AP2A1, APBA1, APBB1, APLP2, APOE, APP, ARHGAP35, BASP1, BSN, CAMK4, CAMSAP2, CAPNS1, CAPRIN1, CAPRIN2, CAV1, CDKL3, CHMP2B, CLSTN1, CLU, COBL, CTTN, DMD, DPYSL4, EEF1A1, EEF2K, EFHD1, ELMO1, EPB41L3, EPS8, GAP43, GAS7, GFAP, GPRIN1, HSPA5, IQGAP1, KIF2A, KIF3A, KIF3C, LAMB1, LRPAP1, MAP1B, MARCKS, MECP2, MINK1, MYO6, MYOC, NCAM1, NEFH, NEFL, NEFM, NLGN3, NR3C1, NRCAM, NRDC, PAFAH1B1, PAK1, PEX14, RAB10, RAB6A, RAB6B, RANBP1, RAP1A, RAP1B, RTN3, RTN4, RUFY3, SNAP25, SNCA, SNCB, SPAG9, SPOCK1, STAU2, SV2A, SYT1, TNR, TOP2B, VGF, YWHAH, ZPR1	80
**Proliferation of neuronal cells**	1.89 × 10^−13^	−0.098	AATK, APBB1, APOE, APP, ATP, BASP1, CAMK4, CAV1, CDC42BPA, CLU, CNPY2, COBL, CPE, EEA1, EPN1, F2, FBLN5, FKBP5, GAL, GAP43, GARS1, GAS7, GFAP, GNAI2, GPRIN1, HMGCS1, IQGAP1, KIF2A, KIF3A, KIF3C, MAP1B, MARCKS, MECP2, NCAM1, NEFH, NEFM, NPY, NRCAM, PAFAH1B1, PAK1, PARP1, PCSK1N, PJA2, RAP1B, RTN4, SET, SLC2A3, SNAP25, SPP1, THBS1, TRPV2, VCAN, VGF, VTI1A, VTN, ZPR1	56
**Synaptic transmission**	3.38 × 10^−13^	1.277	AGRN, AKAP12, APBA1, APOE, APP, ATP, BSN, CAMK4, CLSTN1, DBH, DHCR7, DLG2, DMD, DTNA, EPS8, ERC1, GAL, GNAI2, KIF5A, MECP2, MINK1, MYO6, NCAM1, NLGN3, NPY, NR3C1, NRCAM, PAFAH1B1, PARK7, PRKAR2B, RIMBP2, RTN4, SH3GL1, SNAP25, SNCA, SNCB, SV2A, SV2C, SYT1, SYT5, TNR, UTS2, VGF	43
**Branching of neurons**	3.64 × 10^−13^	0.064	AGRN, APBA1, APLP2, APOE, APP, BASP1, BSN, CAMK4, CAPNS1, CAPRIN1, CAPRIN2, CAV1, CDKL3, CHMP2B, CLSTN1, COBL, DPYSL4, EEF2K, ELMO1, EPS8, GAP43, KIF2A, KIF3A, LRPAP1, MAP1B, MARCKS, MECP2, NCAM1, NEFH, NEFL, NEFM, NLGN3, NR3C1, NRCAM, PAFAH1B1, PAK1, PEX14, RTN4, SNCA, SPAG9, STAU2, SV2A, SYT1, TNR, VGF	45
**Branching of neurites**	6.26 × 10^−13^	−0.134	AGRN, APBA1, APLP2, APOE, APP, BSN, CAMK4, CAPNS1, CAPRIN1, CAPRIN2, CAV1, CDKL3, CHMP2B, CLSTN1, COBL, DPYSL4, EEF2K, ELMO1, EPS8, GAP43, KIF2A, KIF3A, LRPAP1, MAP1B, MARCKS, MECP2, NCAM1, NEFH, NEFL, NEFM, NLGN3, NR3C1, NRCAM, PAFAH1B1, PAK1, PEX14, RTN4, SNCA, SPAG9, STAU2, SV2A, SYT1, TNR, VGF	44
**Morphology of nervous system**	8.27 × 10^−13^		AATK, APBA1, APOE, APP, ATP, CADM4, CAMSAP2, CAV1, DHCR7, DLGAP4, DPYSL4, EEF1A2, EPB41L1, GAL, GAP43, GFAP, GLB1, IGF2R, IQGAP1, KIF2A, KIF5A, KIF5C, LRPAP1, MAP1B, MARCKS, MARCKSL1, MECP2, MYH14, MYO6, MYOC, NAMPT, NCAM1, NEFH, NEFL, NEFM, NLGN3, NRCAM, PAFAH1B1, PAK1, RAB10, RTN4, SLC2A3, SNCA, SNCB, SUN1, TOP2B, TRAPPC9, VAMP2, VTI1A	49
**Dendritic growth/branching**	1.38 × 10^−12^	−0.136	AGRN, APBA1, APLP2, APOE, APP, BSN, CAMK4, CAPNS1, CAPRIN1, CAPRIN2, CAV1, CDKL3, CHMP2B, CLSTN1, COBL, DPYSL4, EEF2K, ELMO1, EPS8, GAP43, KIF3A, MARCKS, MECP2, NEFH, NEFL, NEFM, NLGN3, NR3C1, NRCAM, PAFAH1B1, PAK1, RTN4, SNCA, SPAG9, STAU2, SV2A, SYT1, TNR, VGF	39
**Growth of neurites**	3.8 × 10^−12^	−0.839	AATK, APBB1, APOE, APP, ATP, BASP1, CAMK4, CAV1, CDC42BPA, CNPY2, COBL, EEA1, EPN1, F2, FKBP5, GAL, GAP43, GAS7, GFAP, GNAI2, GPRIN1, HMGCS1, IQGAP1, KIF2A, KIF3A, KIF3C, MAP1B, MARCKS, NCAM1, NEFH, NEFM, NRCAM, PAFAH1B1, PAK1, PARP1, PCSK1N, PJA2, RAP1B, RTN4, SET, SLC2A3, SNAP25, THBS1, TRPV2, VCAN, VGF, VTI1A, ZPR1	48
**Neurotransmission**	4.05 × 10^−12^	1.05	AGRN, AKAP12, APBA1, APOE, APP, ATP, BSN, CAMK4, CLSTN1, CPLX2, DBH, DHCR7, DLG2, DMD, DTNA, EPS8, ERC1, GAL, GNAI2, GPRASP2, KIF5A, MECP2, MINK1, MYH14, MYO6, NCAM1, NLGN3, NPY, NR3C1, NRCAM, PAFAH1B1, PARK7, PRKAR2B, RIMBP2, RTN4, SH3GL1, SNAP25, SNCA, SNCB, SV2A, SV2C, SYT1, SYT5, TNR, UTS2, VAMP2, VGF	47
**Long-term potentiation**	3.87 × 10^−11^	−1.518	APLP2, APOE, APP, ATP, CAMK4, CAPNS1, CDH1, CKAP5, CLSTN1, CPLX2, DBI, DPYSL4, GAL, GFAP, GNAI2, IGF2R, ITPR3, LRPAP1, MECP2, MPP2, NCAM1, NLGN3, NPY, PAK1, PJA2, PPP1R1B, RTN4, SNAP25, SNCA, STAT1, SYNJ1, TNR, VAMP2	33
**Outgrowth of neurons**	4.62 × 10^−11^	−0.488	AATK, APBB1, APOE, APP, ATP, BASP1, CDC42BPA, CLU, CNPY2, EEA1, EPN1, F2, FKBP5, GAL, GAP43, GARS1, GAS7, GFAP, GPRIN1, IQGAP1, KIF2A, KIF3A, KIF3C, MAP1B, MARCKS, MECP2, NCAM1, NEFH, NRCAM, PAFAH1B1, PAK1, PARP1, PCSK1N, PJA2, RAP1B, RTN4, SET, THBS1, TRPV2, VCAN, VTI1A	41
**Quantity of neurons**	4.79 × 10^−11^	−1.5	AGRN, APBB1, APLP2, APOE, APP, ATP, BSN, CAMK4, CLSTN1, CTSD, DNAJC6, DPYSL4, EPS8, F2, GAL, GFAP, GNAI2, HSPB8, HSPD1, KIF5A, KIF5C, LRPAP1, MAP1B, MECP2, MYO6, NCAM1, NEFH, NEFL, NEFM, NLGN3, NPY, NRCAM, OAT, PAFAH1B1, PAK1, SH3GL1, SLC2A3, SMARCE1, SNCA, SNCB, SPP1, SQSTM1, STAM, TNR, TUB, VAMP2, VGF, ZDHHC5	48
**Development of central nervous system**	1.21 × 10^−10^	−1.016	AATK, ALDH1A3, APBB1, APLP2, APOE, APP, ARHGAP35, ATP, ATP5PB, ATP5PF, ATP6AP2, BASP1, COL2A1, COL4A1, CYCS, DBI, DHCR7, DHX30, EEF2K, EGLN1, GAP43, GFAP, GSTP1, HSPA5, HSPB8, IDH1, KIF2A, KIF3A, LDHA, MAP1B, MARCKS, MARCKSL1, MAST1, MECP2, NCAM1, NDRG1, NLGN3, NR3C1, NRCAM, PAFAH1B1, PAK1, PFDN1, QARS1, RPL10, RTN4, SDF4, SH3GL1, SNCA, SNCB, STAM, SUN1, TACC1, THBS1, TMX2, TOP2B, TP53BP2, TRAPPC9, VCAN, VTN, YWHAH, ZPR1	61
**Long-term potentiation of brain**	4.63 × 10^−10^	−2.247	APOE, APP, ATP, CAMK4, CAPNS1, CDH1, CKAP5, GAL, GNAI2, IGF2R, LRPAP1, MECP2, NLGN3, PAK1, PJA2, PPP1R1B, RTN4, SNCA, STAT1, SYNJ1, TNR	21
**Outgrowth of neurites**	8.81 × 10^−10^	−0.879	AATK, APBB1, APOE, APP, ATP, BASP1, CDC42BPA, CNPY2, EEA1, EPN1, F2, FKBP5, GAL, GAP43, GAS7, GFAP, GPRIN1, IQGAP1, KIF2A, KIF3A, KIF3C, MAP1B, MARCKS, NCAM1, NEFH, NRCAM, PAFAH1B1, PAK1, PARP1, PCSK1N, PJA2, RAP1B, RTN4, SET, THBS1, TRPV2, VCAN, VTI1A	38
**Morphology of nervous tissue**	3.26 × 10^−9^		APBA1, APOE, APP, ATP, CADM4, DLGAP4, DPYSL4, EEF1A2, GAL, GAP43, GFAP, IQGAP1, KIF5A, LRPAP1, MAP1B, MECP2, MYH14, MYO6, MYOC, NAMPT, NCAM1, NEFH, NEFL, NEFM, NLGN3, PAFAH1B1, PAK1, RAB10, RTN4, SNCA, SNCB, VAMP2	32

^a^ z-score indicates activation status with negative values indicating inactivation and positive values activation. In cases where no z-score was computed, all associated findings were labeled as showing no increase or decrease; therefore, no data were available to support z-score calculation.

## References

[1] Pirie R.S., Jago R.C., Hudson N.P. (2014). Equine grass sickness. Equine Vet. J..

[2] Sharp N.J.H., Nash A.S., Griffiths I.R. (1984). Feline dysautonomia (the Key-Gaskell syndrome): A clinical and pathological study of forty cases. J. Small Anim. Pract..

[3] Whitwell K.E. (1991). Do hares suffer from grass sickness?. Vet. Rec..

[4] Longshore R.C., O’Brien D.P., Johnson G.C., Grooters A.M., Kroll R.A. (1996). Dysautonomia in dogs: A retrospective study. J. Vet. Intern. Med. Am. Coll. Vet. Intern. Med..

[5] Kik M.J., van der Hage M.H. (1999). Cecal impaction due to dysautonomia in a llama (Lama glama). J. Zoo Wildl. Med. Off. Publ. Am. Assoc. Zoo Vet..

[6] Pruden S.J., McAllister M.M., Schultheiss P.C., O’Toole D., Christensen D.E. (2004). Abomasal emptying defect of sheep may be an acquired form of dysautonomia. Vet. Pathol..

[7] Hahn C.N., Whitwell K.E., Mayhew I.G. (2005). Neuropathological lesions resembling equine grass sickness in rabbits. Vet. Rec..

[8] Clarke K.E., Sorrell S., Breheny C., Jepson R., Adamantos S., Milne E.M., Gunn-Moore D. (2020). Dysautonomia in 53 cats and dogs: Retrospective review of clinical data and outcome. Vet. Rec..

[9] Hahn C.N., Mayhew I.G., de Lahunta A. (2001). Central neuropathology of equine grass sickness. Acta Neuropathol..

[10] McGorum B.C., Davey T., Dosi M.C.M., Keen J.A., Morrison L.R., Pirie R.S., Shaw D.J., Harris J.B. (2025). Equine grass sickness is associated with major abnormalities in the ultrastructure of skeletal neuromuscular junctions. Equine Vet. J..

[11] McGorum B., Pirie R.S., Bano L., Davey T., Harris J., Montecucco C. (2025). Neurotoxic phospholipase A(2): A proposed cause of equine grass sickness and other animal dysautonomias (multiple system neuropathies). Equine Vet. J..

[12] Montecucco C., Bano L. (2024). A possible cause and therapy for equine grass sickness. Toxicon.

[13] Griffiths I.R., Kyriakides E., Smith S., Howie F., Deary A.W. (1993). Immunocytochemical and lectin histochemical study of neuronal lesions in autonomic ganglia of horses with grass sickness. Equine Vet. J..

[14] McGorum B.C., Pirie R.S., Eaton S.L., Keen J.A., Cumyn E.M., Arnott D.M., Chen W., Lamont D.J., Graham L.C., Llavero Hurtado M. (2015). Proteomic Profiling of Cranial (Superior) Cervical Ganglia Reveals Beta-Amyloid and Ubiquitin Proteasome System Perturbations in an Equine Multiple System Neuropathy. Mol. Cell. Proteom. MCP.

[15] McGorum B.C., Kirk J. (2001). Equine dysautonomia (grass sickness) is associated with altered plasma amino acid levels and depletion of plasma sulphur amino acids. Equine Vet. J..

[16] Doxey D.L., Pogson D.M., Milne E.M., Gilmour J.S., Chisholm H.K. (1992). Clinical equine dysautonomia and autonomic neuron damage. Res. Vet. Sci..

[17] Pogson D.M., Doxey D.L., Gilmour J.S., Milne E.M., Chisholm H.K. (1992). Autonomic neurone degeneration in equine dysautonomia (grass sickness). J. Comp. Pathol..

[18] Irizarry R.A., Hobbs B., Collin F., Beazer-Barclay Y.D., Antonellis K.J., Scherf U., Speed T.P. (2003). Exploration, normalization, and summaries of high density oligonucleotide array probe level data. Biostatistics.

[19] Freeman T.C., Goldovsky L., Brosch M., van Dongen S., Maziere P., Grocock R.J., Freilich S., Thornton J., Enright A.J. (2007). Construction, visualisation, and clustering of transcription networks from microarray expression data. PLoS Comput. Biol..

[20] Fruchterman T.M.J., Rheingold E.M. (1991). Graph drawing by force-directed placement. Softw.-Pract. Exp..

[21] Dongen S.V. (2000). Graph Clustering by Flow Simulation.

[22] Brohee S., van Helden J. (2006). Evaluation of clustering algorithms for protein-protein interaction networks. BMC Bioinform..

[23] Huang D.W., Sherman B.T., Lempicki R.A. (2009). Systematic and integrative analysis of large gene lists using DAVID bioinformatics resources. Nat. Protoc..

[24] Huang D.W., Sherman B.T., Lempicki R.A. (2009). Bioinformatics enrichment tools: Paths toward the comprehensive functional analysis of large gene lists. Nucleic Acids Res..

[25] Kramer A., Green J., Pollard J., Tugendreich S. (2014). Causal analysis approaches in Ingenuity Pathway Analysis. Bioinformatics.

[26] Rath S., Sharma R., Gupta R., Ast T., Chan C., Durham T.J., Goodman R.P., Grabarek Z., Haas M.E., Hung W.H.W. (2021). MitoCarta3.0: An updated mitochondrial proteome now with sub-organelle localization and pathway annotations. Nucleic Acids Res..

[27] Wishart T.M., Huang J.P., Murray L.M., Lamont D.J., Mutsaers C.A., Ross J., Geldsetzer P., Ansorge O., Talbot K., Parson S.H. (2010). SMN deficiency disrupts brain development in a mouse model of severe spinal muscular atrophy. Hum. Mol. Genet..

[28] Wishart T.M., Rooney T.M., Lamont D.J., Wright A.K., Morton A.J., Jackson M., Freeman M.R., Gillingwater T.H. (2012). Combining comparative proteomics and molecular genetics uncovers regulators of synaptic and axonal stability and degeneration in vivo. PLoS Genet..

[29] Calderone A., Formenti M., Aprea F., Papa M., Alberghina L., Colangelo A.M., Bertolazzi P. (2016). Comparing Alzheimer’s and Parkinson’s diseases networks using graph communities structure. BMC Syst. Biol..

[30] Karagianni A.E., Summers K.M., Courouce A., Depecker M., McGorum B.C., Hume D.A., Pirie R.S. (2019). The Effect of Race Training on the Basal Gene Expression of Alveolar Macrophages Derived From Standardbred Racehorses. J. Equine Vet. Sci..

[31] O’Brien C.L., Summers K.M., Martin N.M., Carter-Cusack D., Yang Y., Barua R., Dixit O.V.A., Hume D.A., Pavli P. (2024). The relationship between extreme inter-individual variation in macrophage gene expression and genetic susceptibility to inflammatory bowel disease. Hum. Genet..

[32] Finno C.J., Bordbari M.H., Valberg S.J., Lee D., Herron J., Hines K., Monsour T., Scott E., Bannasch D.L., Mickelson J. (2016). Transcriptome profiling of equine vitamin E deficient neuroaxonal dystrophy identifies upregulation of liver X receptor target genes. Free Radic. Biol. Med..

[33] Ramirez A., Heimbach A., Grundemann J., Stiller B., Hampshire D., Cid L.P., Goebel I., Mubaidin A.F., Wriekat A.L., Roeper J. (2006). Hereditary parkinsonism with dementia is caused by mutations in ATP13A2, encoding a lysosomal type 5 P-type ATPase. Nat. Genet..

[34] Tan J., Zhang T., Jiang L., Chi J., Hu D., Pan Q., Wang D., Zhang Z. (2011). Regulation of intracellular manganese homeostasis by Kufor-Rakeb syndrome-associated ATP13A2 protein. J. Biol. Chem..

[35] Zhao L., Rosales C., Seburn K., Ron D., Ackerman S.L. (2010). Alteration of the unfolded protein response modifies neurodegeneration in a mouse model of Marinesco-Sjogren syndrome. Hum. Mol. Genet..

[36] Kwak Y.D., Wang B., Li J.J., Wang R., Deng Q., Diao S., Chen Y., Xu R., Masliah E., Xu H. (2012). Upregulation of the E3 ligase NEDD4-1 by oxidative stress degrades IGF-1 receptor protein in neurodegeneration. J. Neurosci..

[37] Li M., Baumeister P., Roy B., Phan T., Foti D., Luo S., Lee A.S. (2000). ATF6 as a transcription activator of the endoplasmic reticulum stress element: Thapsigargin stress-induced changes and synergistic interactions with NF-Y and YY1. Mol. Cell. Biol..

[38] Zornig M. (1996). Evan GI: Cell cycle: On target with Myc. Curr. Biol..

[39] Keogh M.J., Chinnery P.F. (2015). Mitochondrial DNA mutations in neurodegeneration. Biochim. Biophys. Acta.

[40] Grunewald A., Rygiel K.A., Hepplewhite P.D., Morris C.M., Picard M., Turnbull D.M. (2016). Mitochondrial DNA Depletion in Respiratory Chain-Deficient Parkinson Disease Neurons. Ann. Neurol..

[41] Ronnback A., Pavlov P.F., Mansory M., Gonze P., Marliere N., Winblad B., Graff C., Behbahani H. (2016). Mitochondrial dysfunction in a transgenic mouse model expressing human amyloid precursor protein (APP) with the Arctic mutation. J. Neurochem..

[42] Altimiras F., Uszczynska-Ratajczak B., Camara F., Vlasova A., Palumbo E., Newhouse S., Deacon R.M., Farias L.A., Hurley M.J., Loyola D.E. (2017). Brain Transcriptome Sequencing of a Natural Model of Alzheimer’s Disease. Front. Aging Neurosci..

[43] Pollin M.M., Griffiths I.R. (1992). A review of the primary dysautonomias of domestic animals. J. Comp. Pathol..

[44] Zhou X., Michal J.J., Zhang L., Ding B., Lunney J.K., Liu B., Jiang Z. (2013). Interferon induced IFIT family genes in host antiviral defense. Int. J. Biol. Sci..

[45] Vladimer G.I., Gorna M.W., Superti-Furga G. (2014). IFITs: Emerging Roles as Key Anti-Viral Proteins. Front. Immunol..

[46] Fensterl V., Sen G.C. (2015). Interferon-induced Ifit proteins: Their role in viral pathogenesis. J. Virol..

[47] Lattin J.E., Schroder K., Su A.I., Walker J.R., Zhang J., Wiltshire T., Saijo K., Glass C.K., Hume D.A., Kellie S. (2008). Expression analysis of G Protein-Coupled Receptors in mouse macrophages. Immunome Res..

[48] Takeuchi O., Akira S. (2010). Pattern recognition receptors and inflammation. Cell.

[49] Stawowczyk M., Van Scoy S., Kumar K.P., Reich N.C. (2011). The interferon stimulated gene 54 promotes apoptosis. J. Biol. Chem..

[50] Smith E.L., Schuchman E.H. (2008). The unexpected role of acid sphingomyelinase in cell death and the pathophysiology of common diseases. FASEB J..

[51] Candalija A., Cubi R., Ortega A., Aguilera J., Gil C. (2014). Trk receptors need neutral sphingomyelinase activity to promote cell viability. FEBS Lett..

[52] Herdman C., Tremblay M.G., Mishra P.K., Moss T. (2014). Loss of Extended Synaptotagmins ESyt2 and ESyt3 does not affect mouse development or viability, but in vitro cell migration and survival under stress are affected. Cell Cycle.

[53] Hume D.A., Summers K.M., Raza S., Baillie J.K., Freeman T.C. (2010). Functional clustering and lineage markers: Insights into cellular differentiation and gene function from large-scale microarray studies of purified primary cell populations. Genomics.

[54] Summers K.M., Raza S., van Nimwegen E., Freeman T.C., Hume D.A. (2010). Co-expression of FBN1 with mesenchyme-specific genes in mouse cell lines: Implications for phenotypic variability in Marfan syndrome. Eur. J. Hum. Genet..

[55] Mabbott N.A., Kenneth Baillie J., Kobayashi A., Donaldson D.S., Ohmori H., Yoon S.O., Freedman A.S., Freeman T.C., Summers K.M. (2011). Expression of mesenchyme-specific gene signatures by follicular dendritic cells: Insights from the meta-analysis of microarray data from multiple mouse cell populations. Immunology.

[56] Chen G., Gharib T.G., Huang C.C., Taylor J.M., Misek D.E., Kardia S.L., Giordano T.J., Iannettoni M.D., Orringer M.B., Hanash S.M. (2002). Discordant protein and mRNA expression in lung adenocarcinomas. Mol. Cell. Proteom..

[57] Ghazalpour A., Bennett B., Petyuk V.A., Orozco L., Hagopian R., Mungrue I.N., Farber C.R., Sinsheimer J., Kang H.M., Furlotte N. (2011). Comparative analysis of proteome and transcriptome variation in mouse. PLoS Genet..

[58] Haider S., Pal R. (2013). Integrated analysis of transcriptomic and proteomic data. Curr. Genom..

[59] Karagianni A.E., Kurian D., Cillan-Garcia E., Eaton S.L., Wishart T.M., Pirie R.S. (2022). Training associated alterations in equine respiratory immunity using a multiomics comparative approach. Sci. Rep..

[60] Karagianni A.E., Richard E.A., Toquet M.P., Hue E.S., Courouce-Malblanc A., McGorum B., Kurian D., Aguilar J., Mazeri S., Wishart T.M. (2024). Distinct Molecular Profiles Underpin Mild-To-Moderate Equine Asthma Cytological Profiles. Cells.

[61] Loeb J. (2022). Biobank samples could help solve equine grass sickness. Vet. Rec..

[62] Wells B. (2024). Update on the equine grass sickness biobank, database and research project. Vet. Rec..

