# Supplementary material for: The Transcriptomic and Proteomic Molecular Signatures of Equine Multiple-System Neuropathy (Grass Sickness)
Source: Cells. 2026 Jul 24;15(15):1328. doi: 10.3390/cells15151328 (PMC13464567; doi:10.3390/cells15151328)
Supplement: Supplementary file 1 [file cells-15-01328-s001.zip › Table S1. GSEA analysis of transcriptomic data.pdf]

Online Resource 1.

Results of GSEA analysis

A. Heat map of the top 50 features for each phenotype.

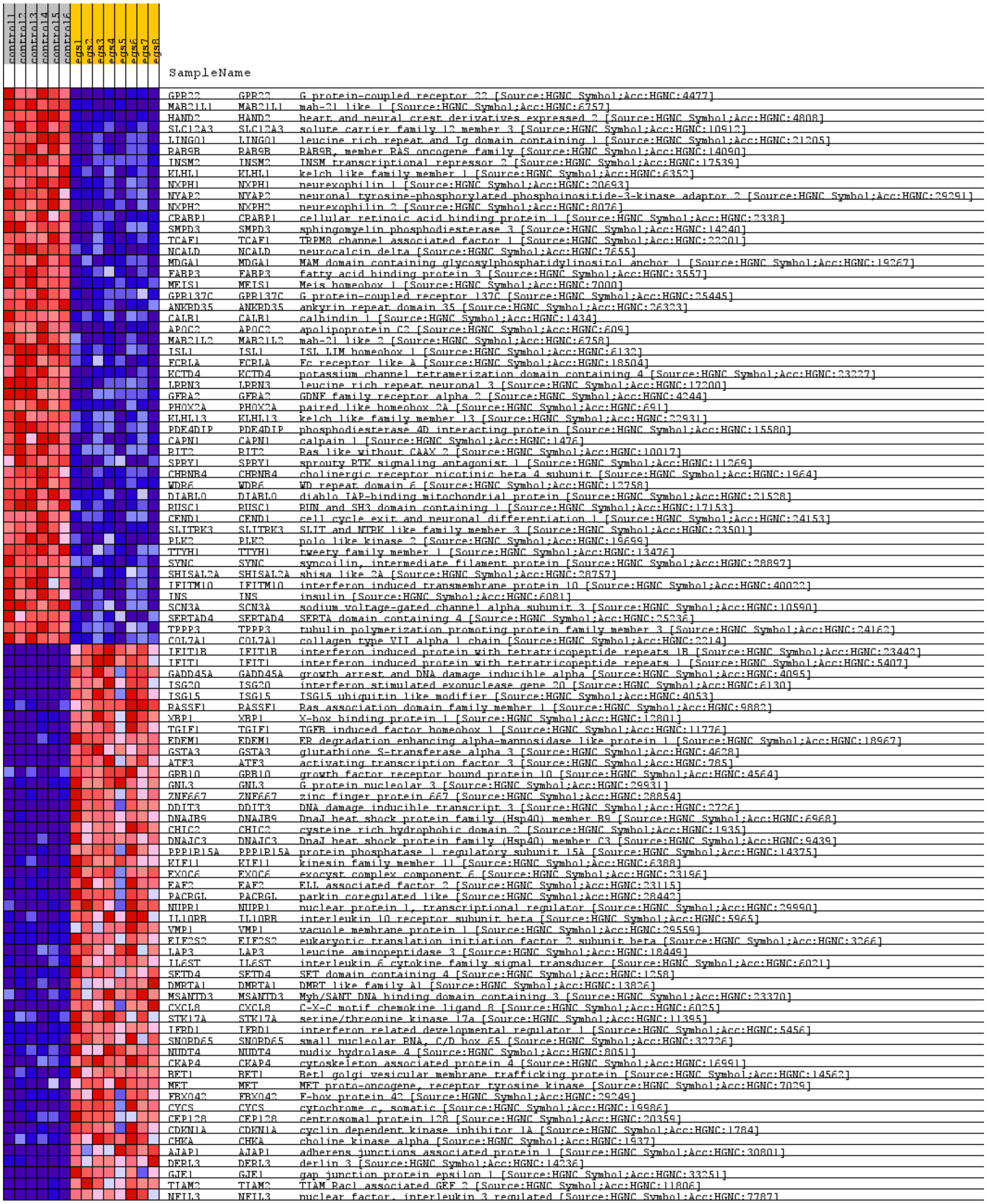

B. Gene sets enriched in controls

	GS follow link to MSigDB	GS DETAILS	SIZE	ES	NES	NOM p-val	FDR q-val	FWER p-val	RANK AT MAX	LEADING EDGE
1	HALLMARK_APICAL_SURFACE	Details ...	27	0.52	1.75	0.000	0.055	0.064	822	tags=22%, list=7%, signal=24%
2	HALLMARK_CHOLESTEROL_HOMEOSTASIS	Details ...	66	0.41	1.62	0.002	0.134	0.246	1838	tags=42%, list=16%, signal=50%
3	HALLMARK_PANCREAS_BETA_CELLS	Details ...	22	0.43	1.49	0.042	0.272	0.514	1079	tags=27%, list=10%, signal=30%
4	HALLMARK_OXIDATIVE_PHOSPHORYLATION	Details ...	179	0.40	1.43	0.081	0.290	0.632	3177	tags=47%, list=28%, signal=64%
5	HALLMARK_FATTY_ACID_METABOLISM	Details ...	123	0.34	1.43	0.097	0.248	0.651	2705	tags=33%, list=24%, signal=43%
6	HALLMARK_KRAS_SIGNALING_DN	Details ...	106	0.37	1.41	0.030	0.230	0.684	2208	tags=33%, list=19%, signal=41%
7	HALLMARK_MYOGENESIS	Details ...	143	0.32	1.39	0.084	0.216	0.715	3138	tags=38%, list=28%, signal=53%
8	HALLMARK_APICAL_JUNCTION	Details ...	140	0.35	1.37	0.115	0.220	0.762	2224	tags=29%, list=20%, signal=36%
9	HALLMARK_WNT_BETA_CATENIN_SIGNALING	Details ...	34	0.37	1.35	0.126	0.214	0.784	1591	tags=26%, list=14%, signal=31%
10	HALLMARK_HEDGEHOG_SIGNALING	Details ...	27	0.38	1.35	0.117	0.197	0.791	1394	tags=30%, list=12%, signal=34%
11	HALLMARK_PEROXISOME	Details ...	81	0.28	1.24	0.158	0.310	0.913	3862	tags=48%, list=34%, signal=73%
12	HALLMARK_NOTCH_SIGNALING	Details ...	22	0.36	1.22	0.188	0.300	0.922	4613	tags=64%, list=41%, signal=107%
13	HALLMARK_ADIPOGENESIS	Details ...	171	0.22	1.10	0.281	0.446	0.982	3223	tags=36%, list=28%, signal=49%
14	HALLMARK_XENOBIOTIC_METABOLISM	Details ...	144	0.21	1.04	0.371	0.506	0.991	2792	tags=31%, list=25%, signal=40%
15	HALLMARK_BILE_ACID_METABOLISM	Details ...	77	0.24	1.01	0.433	0.525	0.994	2223	tags=25%, list=20%, signal=30%
16	HALLMARK_UV_RESPONSE_DN	Details ...	119	0.26	0.93	0.530	0.620	0.997	1719	tags=19%, list=15%, signal=23%
17	HALLMARK_MITOTIC_SPINDLE	Details ...	152	0.14	0.74	0.919	0.862	1.000	3294	tags=26%, list=29%, signal=37%
18	HALLMARK_ANGIOGENESIS	Details ...	27	0.21	0.55	0.971	0.958	1.000	1394	tags=15%, list=12%, signal=17%

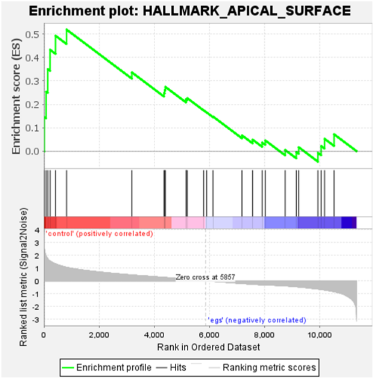

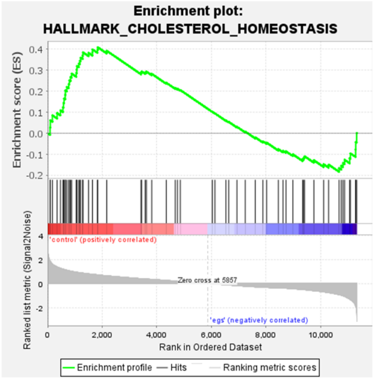

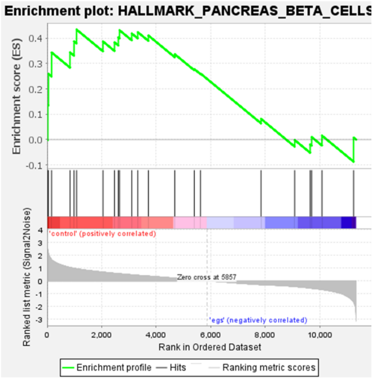

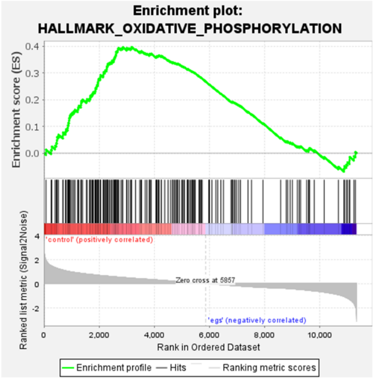

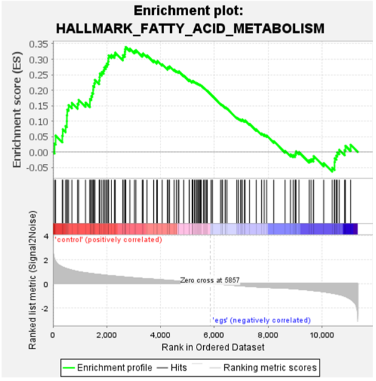

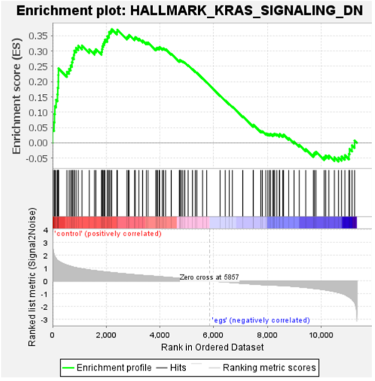

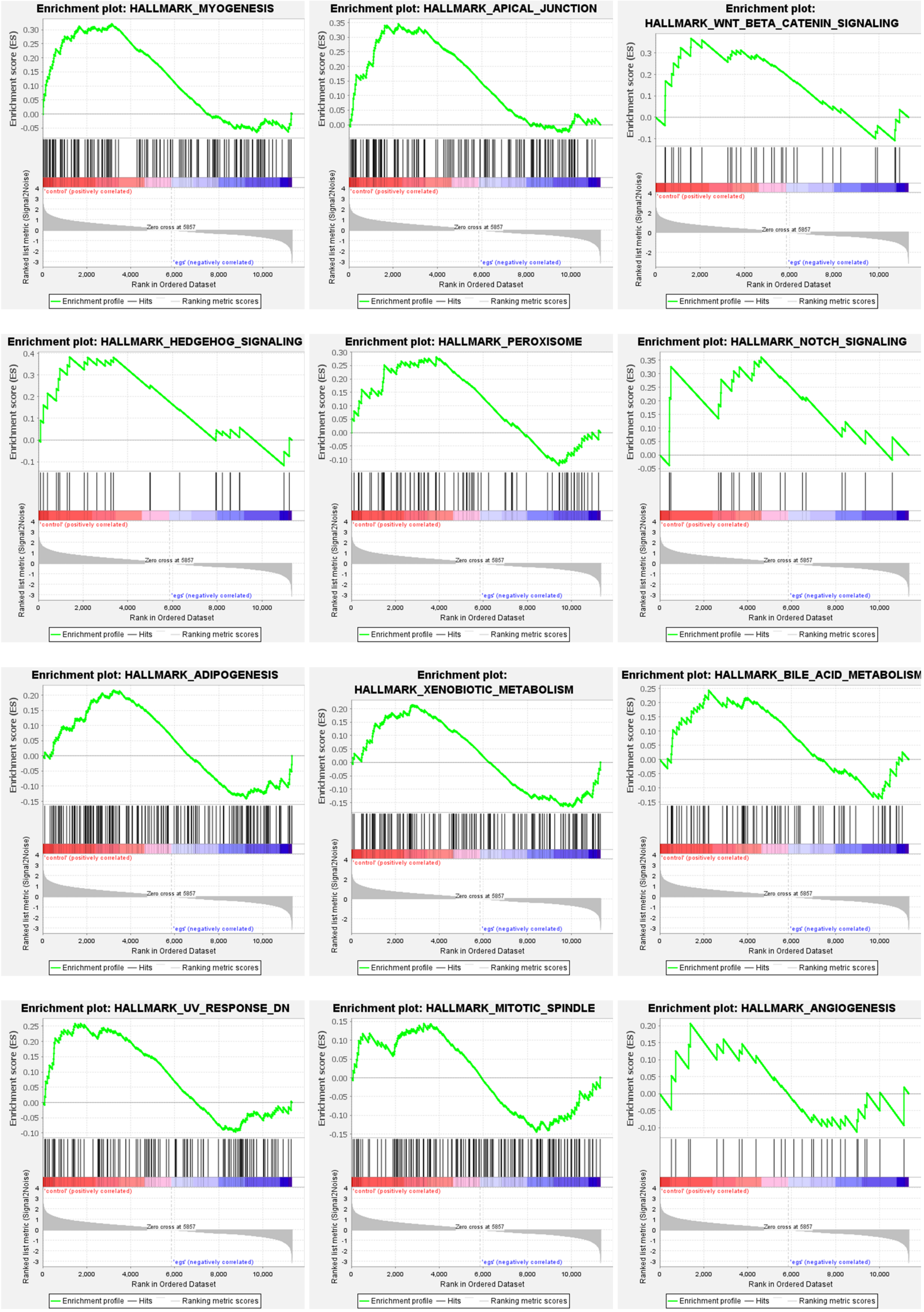
Enrichment plot: HALLMARK_MYOGENESIS
Enrichment plot: HALLMARK_APICAL_JUNCTION
Enrichment plot: HALLMARK_WNT_BETA_CATENIN_SIGNALING
Enrichment plot: HALLMARK_HEDGEHOG_SIGNALING
Enrichment plot: HALLMARK_PEROXISOME
Enrichment plot: HALLMARK_NOTCH_SIGNALING
Enrichment plot: HALLMARK_ADIPOGENESIS
Enrichment plot: HALLMARK_XENOBIOTIC_METABOLISM
Enrichment plot: HALLMARK_BILE_ACID_METABOLISM
Enrichment plot: HALLMARK_UV_RESPONSE_DN
Enrichment plot: HALLMARK_MITOTIC_SPINDLE
Enrichment plot: HALLMARK_ANGIOGENESIS
Enrichment score (ES)
Ranked list metric (Signal2Noise)
'control' (positively correlated)
Zero cross at 5857
'egs' (negatively correlated)
Rank in Ordered Dataset
Enrichment profile
Hits
Ranking metric scores

C. Gene sets enriched in EGS

	GS follow link to MSigDB	GS DETAILS	SIZE	ES	NES	NOM p-val	FDR q-val	FWER p-val	RANK AT MAX	LEADING EDGE
1	HALLMARK_UNFOLDED_PROTEIN_RESPONSE	Details ...	104	-0.61	-1.97	0.002	0.004	0.003	2000	tags=56%, list=18%, signal=67%
2	HALLMARK_APOPTOSIS	Details ...	127	-0.51	-1.95	0.000	0.003	0.005	1641	tags=41%, list=14%, signal=47%
3	HALLMARK_MYC_TARGETS_V2	Details ...	54	-0.58	-1.80	0.018	0.023	0.044	1266	tags=43%, list=11%, signal=48%
4	HALLMARK_INTERFERON_GAMMA_RESPONSE	Details ...	159	-0.62	-1.71	0.010	0.047	0.113	2093	tags=58%, list=18%, signal=70%
5	HALLMARK_TNFA_SIGNALING_VIA_NFKB	Details ...	154	-0.62	-1.68	0.008	0.054	0.156	2678	tags=68%, list=24%, signal=87%
6	HALLMARK_INTERFERON_ALPHA_RESPONSE	Details ...	86	-0.67	-1.67	0.013	0.050	0.173	2073	tags=62%, list=18%, signal=75%
7	HALLMARK_MYC_TARGETS_V1	Details ...	181	-0.44	-1.66	0.020	0.045	0.183	2636	tags=44%, list=23%, signal=57%
8	HALLMARK_P53_PATHWAY	Details ...	151	-0.36	-1.65	0.016	0.045	0.195	1641	tags=31%, list=14%, signal=36%
9	HALLMARK_IL6_JAK_STAT3_SIGNALING	Details ...	55	-0.60	-1.64	0.008	0.042	0.200	2392	tags=56%, list=21%, signal=71%
10	HALLMARK_ALLOGRAFT_REJECTION	Details ...	146	-0.45	-1.60	0.024	0.053	0.260	2113	tags=44%, list=19%, signal=53%
11	HALLMARK_MTORC1_SIGNALING	Details ...	177	-0.39	-1.53	0.078	0.083	0.389	1390	tags=31%, list=12%, signal=35%
12	HALLMARK_HYPOXIA	Details ...	160	-0.34	-1.49	0.016	0.108	0.486	1205	tags=24%, list=11%, signal=27%
13	HALLMARK_TGF_BETA_SIGNALING	Details ...	46	-0.42	-1.46	0.034	0.119	0.540	2174	tags=41%, list=19%, signal=51%
14	HALLMARK_INFLAMMATORY_RESPONSE	Details ...	131	-0.46	-1.45	0.067	0.120	0.571	2129	tags=44%, list=19%, signal=54%
15	HALLMARK_IL2_STAT5_SIGNALING	Details ...	149	-0.37	-1.44	0.029	0.124	0.602	1948	tags=36%, list=17%, signal=43%
16	HALLMARK_ANDROGEN_RESPONSE	Details ...	83	-0.37	-1.43	0.062	0.123	0.621	2052	tags=36%, list=18%, signal=44%
17	HALLMARK_HEME_METABOLISM	Details ...	152	-0.27	-1.38	0.053	0.167	0.738	1379	tags=20%, list=12%, signal=22%
18	HALLMARK_COMPLEMENT	Details ...	148	-0.36	-1.37	0.074	0.163	0.745	2398	tags=45%, list=21%, signal=57%
19	HALLMARK_G2M_CHECKPOINT	Details ...	138	-0.32	-1.31	0.149	0.220	0.835	2485	tags=36%, list=22%, signal=45%
20	HALLMARK_GLYCOLYSIS	Details ...	159	-0.26	-1.30	0.058	0.225	0.848	1205	tags=19%, list=11%, signal=21%
21	HALLMARK_PI3K_AKT_MTOR_SIGNALING		82	-0.31	-1.28	0.174	0.236	0.881	1738	tags=27%, list=15%, signal=31%
22	HALLMARK_UV_RESPONSE_UP		122	-0.27	-1.20	0.212	0.330	0.946	816	tags=17%, list=7%, signal=18%
23	HALLMARK_ESTROGEN_RESPONSE_LATE		140	-0.23	-1.16	0.184	0.364	0.967	1110	tags=16%, list=10%, signal=17%
24	HALLMARK_E2F_TARGETS		160	-0.27	-1.16	0.295	0.349	0.967	2577	tags=34%, list=23%, signal=43%
25	HALLMARK_ESTROGEN_RESPONSE_EARLY		135	-0.24	-1.16	0.213	0.341	0.968	1478	tags=21%, list=13%, signal=24%
26	HALLMARK_REACTIVE_OXYGEN_SPECIES_PATHWAY		44	-0.27	-1.09	0.352	0.413	0.983	1623	tags=34%, list=14%, signal=40%
27	HALLMARK_PROTEIN_SECRETION		86	-0.29	-1.06	0.434	0.448	0.990	2509	tags=34%, list=22%, signal=43%
28	HALLMARK_KRAS_SIGNALING_UP		154	-0.25	-1.04	0.395	0.454	0.993	2285	tags=31%, list=20%, signal=38%
29	HALLMARK_COAGULATION		100	-0.28	-0.92	0.621	0.621	0.999	3128	tags=45%, list=28%, signal=62%
30	HALLMARK_DNA_REPAIR		119	-0.20	-0.92	0.533	0.601	0.999	2775	tags=28%, list=24%, signal=36%
31	HALLMARK_EPITHELIAL_MESENCHYMAL_TRANSITION		174	-0.28	-0.77	0.727	0.800	1.000	2560	tags=39%, list=23%, signal=49%
32	HALLMARK_SPERMATOGENESIS		79	-0.17	-0.69	0.888	0.867	1.000	2847	tags=32%, list=25%, signal=42%

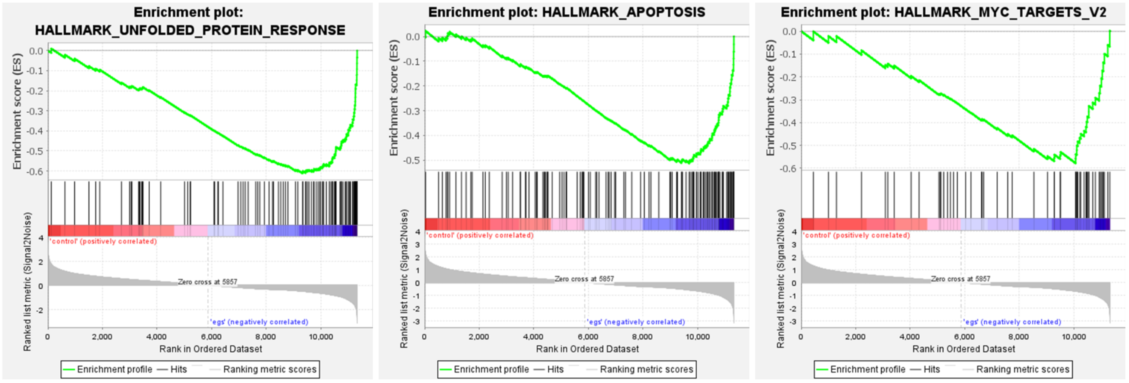

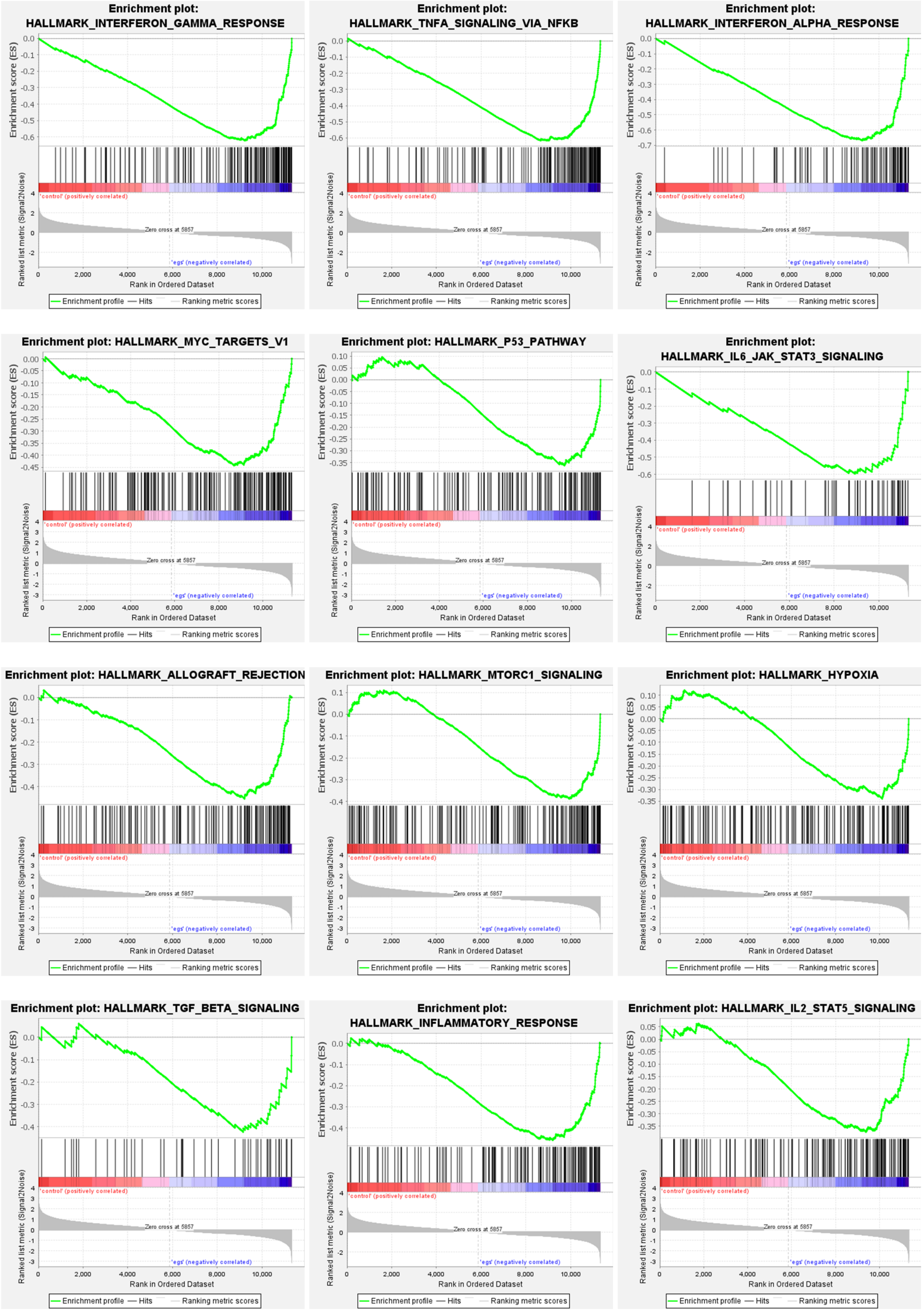
Enrichment plot: HALLMARK_INTERFERON_GAMMA_RESPONSE
Enrichment plot: HALLMARK_TNFA_SIGNALING_VIA_NFKB
Enrichment plot: HALLMARK_INTERFERON_ALPHA_RESPONSE
Enrichment plot: HALLMARK_MYC_TARGETS_V1
Enrichment plot: HALLMARK_P53_PATHWAY
Enrichment plot: HALLMARK_IL6_JAK_STAT3_SIGNALING
Enrichment plot: HALLMARK_ALLOGRAFT_REJECTION
Enrichment plot: HALLMARK_MTORC1_SIGNALING
Enrichment plot: HALLMARK_HYPOXIA
Enrichment plot: HALLMARK_TGF_BETA_SIGNALING
Enrichment plot: HALLMARK_INFLAMMATORY_RESPONSE
Enrichment plot: HALLMARK_IL2_STAT5_SIGNALING
Enrichment score (ES)
Ranked list metric (Signal2Noise)
'control' (positively correlated)
Zero cross at 5857
'egs' (negatively correlated)
Rank in Ordered Dataset
Enrichment profile
Hits
Ranking metric scores

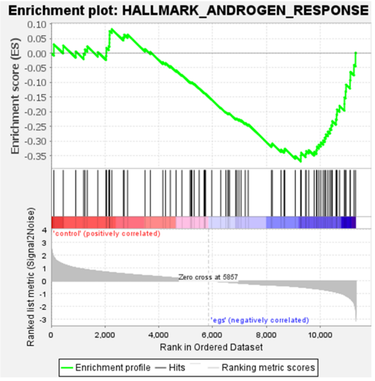
Enrichment plot: HALLMARK_ANDROGEN_RESPONSE
Enrichment score (ES)
'control' (positively correlated)
Zero cross at 5857
'egs' (negatively correlated)
Ranked list metric (Signal2Noise)
Rank in Ordered Dataset
Enrichment profile
Hits
Ranking metric scores

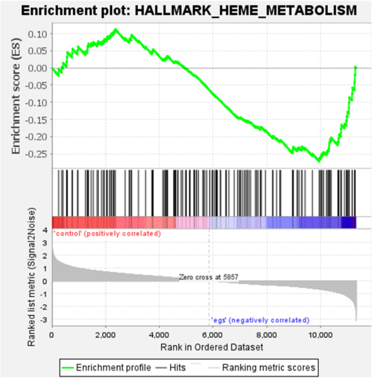
Enrichment plot: HALLMARK_HEME_METABOLISM
Enrichment score (ES)
'control' (positively correlated)
Zero cross at 5857
'egs' (negatively correlated)
Ranked list metric (Signal2Noise)
Rank in Ordered Dataset
Enrichment profile
Hits
Ranking metric scores

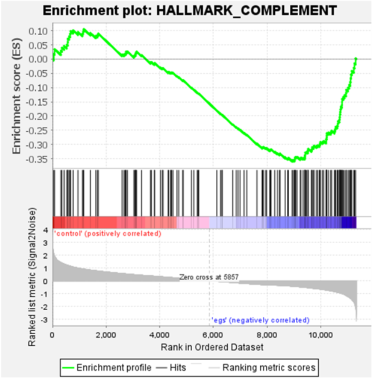
Enrichment plot: HALLMARK_COMPLEMENT
Enrichment score (ES)
'control' (positively correlated)
Zero cross at 5857
'egs' (negatively correlated)
Ranked list metric (Signal2Noise)
Rank in Ordered Dataset
Enrichment profile
Hits
Ranking metric scores

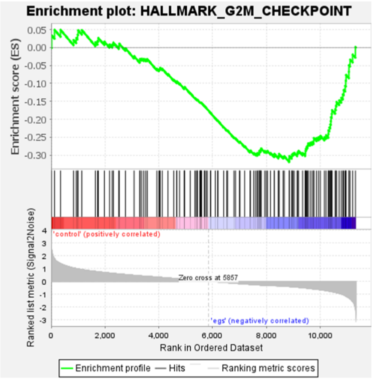
Enrichment plot: HALLMARK_G2M_CHECKPOINT
Enrichment score (ES)
'control' (positively correlated)
Zero cross at 5857
'egs' (negatively correlated)
Ranked list metric (Signal2Noise)
Rank in Ordered Dataset
Enrichment profile
Hits
Ranking metric scores

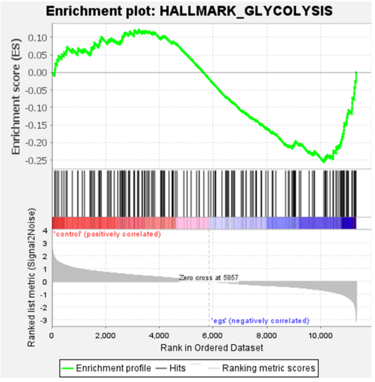
Enrichment plot: HALLMARK_GLYCOLYSIS
Enrichment score (ES)
'control' (positively correlated)
Zero cross at 5857
'egs' (negatively correlated)
Ranked list metric (Signal2Noise)
Rank in Ordered Dataset
Enrichment profile
Hits
Ranking metric scores
